# Bacterial phylotypes associated with rock-dwelling *Umbilicaria* Lichens from Arctic/Subarctic areas in North America and Northern Europe

**DOI:** 10.1007/s00300-024-03303-3

**Published:** 2024-10-21

**Authors:** Zichen He, Takeshi Naganuma, Merry Sailonga Faluaburu, Ryosuke Nakai, Hiroshi Kanda, Masaki Uchida, Satoshi Imura, Martin W. Hahn

**Affiliations:** 1 Graduate School of Integrated Sciences for Life, Hiroshima University, Higashi-Hiroshima, 739-8528 Japan; 2 Graduate School of Biosphere Science, Hiroshima University, Higashi-Hiroshima, 739-8528 Japan; 3 Present Address: Solomon Islands National University, Kukum Highway, P. O. Box R311, Honiara, Solomon Islands; 4 Bioproduction Research Institute, National Institute of Advanced Industrial Science and Technology, Sapporo, 062-8517 Japan; 5 National Institute of Polar Research, 10-3 Midori-Cho, Tachikawa, 190-8518 Japan; 6 Research Department for Limnology, Universität Innsbruck, 5310 Mondsee, Austria

**Keywords:** *Umbilicaria*, V3-V4 region, OTUs, Biogeography, Metabolism, Host-associated bacteria

## Abstract

The diversity of bacteria associated with lichens has received increasing attention. However, studies based on next-generation sequencing of microbiomes have not yet been conducted in the Arctic and Subarctic regions. In this study, rock-dwelling lichens belonging to the Umbilicariaceae family were sampled from the Arctic and Subarctic biological zones. The primary research purpose was to undertake a comparative investigation of the bacterial composition and diversity, identify potential indicators, and explore their potential metabolic pathways. 18S rRNA gene sequences of the fungal partner belonging to the genus *Umbilicaria* (Ascomycota) and the algal partner affiliated with the lineage *Trebouxia* (Chlorophyta). Comparing *Umbilicaria* spp. with a previous study in the Antarctic zone, the fungal partners were more inclined to cluster by sampling region. Operational taxonomic units (OTUs) were established based on a predetermined similarity threshold for V3-V4 sequences, which were ascribed to 19 bacterial phyla, and ten of them were consistently present in all samples. The most distinct zonal indicator genera based on OTU frequencies from Arctic and Subarctic lichens were *Capsulimonas* (Armatimonadota) and *Jatrophihabitans* (Actinomycota), respectively. Although the Subarctic zone had higher biodiversity and species richness based on alpha-diversity, the beta-diversity showed that the main species of bacterial communities were not significantly different, and the predictions of metabolic pathways based on the bacterial microbiome in lichen samples from the two zones were similar. These findings provide evidence that the geographical and/or bioclimatic environment and the different lichen-forming fungal species mainly and partially influence bacterial microbiomes and metabolic pathways.

**Supplementary Information:**

The online version contains supplementary material available at 10.1007/s00300-024-03303-3.

## Introduction

Lichens serve as a food source for the Arctic and Subarctic animals. The reindeer lichen (*Cladonia rangiferina*) is a crucial winter diet for reindeer (or caribou, *Rangifer tarandus*) and benefits local people through reindeer for their food and clothing (Llano 1956; Richardson and Young 1977). Reindeer may also eat rock tripe (*Umbilicaria*, phylum Ascomycota) lichens, as implied by the absence of *Umbilicaria* in reindeer-feeding fields (van der Wal et al. 2001). Ethnolichenologically, among the edible lichens, *Umbilicaria* rock tripes have been used as emergency or famine food by people in the far north and as cuisine in Japan (Angier 1974). The harvesting of cliff-dwelling *Umbilicaria* is depicted in a *ukiyo-e* woodprint (Utagawa 1860).

Rock-dwelling lichens, known as rock tripes, belong to the family Umbilicariaceae (phylum Ascomycota, class Lecanoromycetes, order Umbilicariales) (Smith et al. 2020). The National Center for Biotechnology Information Taxonomy Browser lists more than 70 species in the genus *Umbilicaria*, which has eight subgenera (Davydov et al. 2017; Schoch et al. 2020). According to the occurrence data retrieved from the Consortium of North American Lichen Herbaria, 109 of 751 occurrences of *Umbilicaria*, ca. 14.5%, are in the Arctic and Subarctic zones (usually, Arctic is defined as north of the Arctic Circle (> 66°34′N) and Subarctic as between 50°N and the Arctic Circle.). As of March 05, 2023, a literature search with the keywords “*Umbilicaria* + Arctic − Antarctic” resulted in 15 hits by SCOPUS, one of which reports a draft genome of an endosymbiotic bacterium of an Arctic *Umbilicaria* sp. (Lee et al. 2012). Although the presence of genuine endosymbiotic bacteria in lichens has not yet been explicitly proven, the association (not necessarily symbiosis) of bacteria with lichens has been a topic in microbiology and lichenology, particularly in the era of DNA-based microbiomics using next-generation sequencing (Bates et al. 2011; Greshake Tzovaras et al. 2020; He and Naganuma 2022).

Next-generation sequencing-based microbiomics have been conducted with *Umbilicaria americana* and *Umbilicaria phaea* from Colorado (USA) (Bates et al. 2011), and *Umbilicaria pustulata* from Sardinia (Italy) and Saarland (Germany) (Greshake Tzovaras et al. 2020). However, no such studies have been conducted on Arctic *Umbilicaria*. We previously reported the diversity and composition of bacteria associated with Antarctic *Umbilicaria* (Faluaburu et al. 2019; He et al. 2022) previously. The current study reports the microbiomic phylotypes, or operational taxonomic units (OTUs), based on the V3-V4 region sequences of the bacteria associated with Arctic *Umbilicaria* and compares them with those associated with Antarctic *Umbilicaria*. The similarities and differences between the northern and southern circumpolar regions have been discussed.

## Materials and methods

### Sampling of rock-dwelling *Umbilicaria* Lichens

**Fig. 1:**
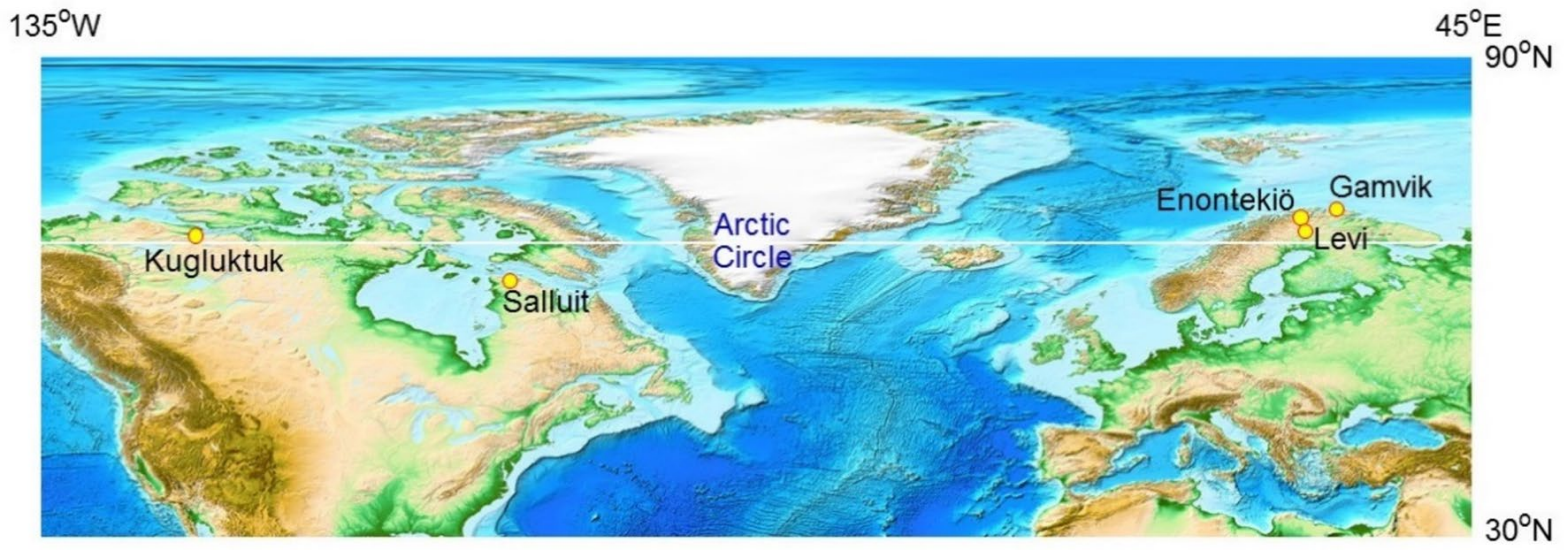
Geographical distribution of rock tripe lichen sampling sites in Kugluktuk (Canada), Salluit (Canada), Enontekiö (Finland), Levi (Finland), and Gamvik (Norway). The distance between Kugluktuk and Gamvik is calculated as approximately 4,353 km. The map image is adopted from the ETOPO1 Global Relief Model (NOAA National Geophysical Data Center 2009)

Lichen specimens presumed to belong to the genus *Umbilicaria* were sampled at Arctic sites in Kugluktuk (Canada), Enontekiö (Finland), Levi (Finland), and Gamvik (Norway), as well as from Subarctic sites in Salluit (Canada), as shown in Fig. 1. Geographical coordinates and altitudes of the sampling sites are listed in Table 1. The distance between Kugluktuk and Gamvik is approximately 4,353 km, as calculated using the Great Circle Calculator (Williams 2003).

**Table 1:** Sampling sites where rock tripe lichens are found in fellfield environments in Kugluktuk (Canada), Gamvik (Norway), Enontekiö (Finland), Kittilä (Finland), and Salluit (Canada)

Zone	Region	Area	Latitude	Longitude	Altitude (m)	Sample Code
Arctic	Canadian Arctic	Kugluktuk	67°48′ 37.0″ N	115°12′ 22.0″ W	10	N01, N02
	Norway	Gamvik	71°02′ 18.5″ N	28°10′ 56.9″ E	70	N03, N04
	Finland	Enontekiö	68°27′ 31.4″ N	23°25′ 26.4″ E	342	N05, N06
		Levi	67°55′ 25.2″ N	24°25′ 39.6″ E	275	N07, N08, N09
Subarctic	Canadian Subarctic	Salluit	62˚12′ 10.5″ N	75˚37′ 32.7″ W	92	N10
			62˚12′ 02.9″ N	75˚ 37′ 38.4″ W	73	N11
			62˚12′ 10.9″ N	75˚38′ 19.8″ W	10	N12
			62˚12′ 05.0″ N	75˚39′ 00.3″ W	35	N13
			62˚12′ 58.7″ N	75˚37′ 54.9″ W	136	N14
			62˚13′ 00.0″ N	75˚37′ 56.8″ W	125	N15
			62˚11′ 24.9″ N	75˚38′ 35.2″ W	72	N16
			62˚11′ 24.4″ N	75˚38′ 38.5″ W	69	N17
			62˚11′ 42.1″ N	75˚38′ 49.1″ W	90	N18

Geographical coordinates and elevations of the Salluit sites were determined with GPSMAP62S (Garmin, Olathe, Kansas, USA)

Kugluktuk, previously known as coppermine, is where the Coppermine River enters the Coronation Gulf. A rocky and frequently desolated Canadian Shield dominates the surrounding area. Kugluktuk features wet summers and cold, dry winters with a daily minimum temperature of −32 °C in February, a daily maximum temperature of 15.6 °C in July, and an annual mean temperature of −10.3 °C (Government of Canada 2020). The lichens in Kugluktuk were sampled on September 3, 2009, stored in the herbarium of the National Institute of Polar Research (NIPR, Japan), and transferred to Hiroshima University on January 11, 2019.

Lichens from Gamvik, Enontekiö, and Levi were sampled on August 8, 10, and 11, 2016, respectively, and transferred to Hiroshima University on August 29, 2016. Gamvik is located on Nordkinn Peninsula, the northernmost part of Norway as well as mainland Europe, and has a boreal climate with a daily minimum temperature of −16.0°C in December, a daily maximum temperature of 30.8°C in June, and a mean temperature of 2.8°C during September 2021 to September 2022 (Norwegian Meteorological Institute and the Norwegian Broadcasting Corporation 2007). Enontekiö and Levi are located in the Finnish Lapland region in the outermost northwestern part of Finland. At Enontekiö, annual minimum, annual maximum, and annual mean temperatures in 1991–2021 are −15.2°C, 11.2°C, and −0.6°C, respectively (Climate-Data.org 2012a), while those at Kittilä, approximately 20 km from Levi, are −11.7°C, 17.9°C, and 0.6°C, respectively (Climate-Data.org 2012b). The three areas are within a 400 km range within the mainland European Arctic.

Salluit is located close to the Hudson Strait, which links the Labrador Sea to Hudson Bay in Canada. Lichens in Salluit were sampled on 10–13 July 2017 and transferred immediately to Hiroshima University. Annual minimum, annual maximum, and annual mean temperatures during 1985–2025 are averaged −28°C, 12°C, and −8°C, respectively (Time and Date AS 1997).

During field sampling, lichen thalli were meticulously removed using a surgical blade that had been sterilized with a flame and collected in clean paraffin paper containers at Kugluktuk; pre-sterilized 50-mL centrifuge tubes at Gamvik, Enontekiö, and Levi; and pre-sterilized Nasco (Atkinson, USA) whirl–pak packages that had been previously sterilized at Salluit. Each lichen thallus (approximately 1 g) was individually packaged to ensure that only one *Umbilicaria* spp. was sampled. At Hiroshima University, the thalli were frozen at -30°C until DNA extraction.

### Extraction of bulk DNA from thalli of the lichen samples

The individually packaged lichen thalli collected from each specified sampling position were washed with autoclaved Milli-Q ultrapure water, cut into less than 5 × 5-mm pieces, and ground for bulk DNA extraction, as described in previous studies (Faluaburu et al. 2019; He et al. 2022). The purity and quantity of the extracted DNA were assessed with a Thermo Fisher Scientific (Waltham, MA, USA) produced NanoDrop 2000c. Before the polymerase chain reaction (PCR) amplification, the DNA samples were then securely stored at a temperature of -20 °C.

### Amplification and sequencing of fungal/Algal 18S rRNA gene

Genomic DNAs extracted from the lichen samples was used to amplify the sequences of the fungal/algal 18S rRNA gene (near-full-length) using TaKaRa Bio (Kusatsu, Japan), which produced a PCR kit, TaKaRa Ex Taq (Mg^2+^ plus Buffer). The PCR machines used in this study included three TaKaRa Bio machines, a “TaKaRa PCR Thermal Cycler Dice Touch TP350”, and two “TaKaRa PCR Thermal Cycler PERSONAL”s. The primer sequences and target sequences are listed in Table 2. For the process of fungal 18S rRNA genes amplification, the PCR protocol comprised an initial denaturation phase, 30 cycles involving denaturation/annealing/extension, and a final extension step at 95°C for 5 min, 95°C for 45 s/61°C for 45 s/72°C for 80 s, and 72°C for 12 min, respectively. The same protocol was used for algal 18S rRNA gene amplification but annealing at 53°C (Faluaburu et al. 2019). The PCR amplification products of the fungal/algal 18S rRNA gene were purified using High Pure PCR Product Purification Kit (Roche, Basel, Switzerland) and Sanger sequenced at the Department of Gene Science, Natural Science Center for Basic Research and Development (N-BARD) (He et al. 2022).

**Table 2:** List of primers with target sequences in this study. F and R mean forward and reverse primer

Target sequence	Primer designation	F/R	Length (-mer)	5′ → 3′	Expected product size	References
Fungal 18S rRNA gene	NS17UCB	F	19	CATGTCTAAGTTTAAGCAA	2.0 kbp	(Gargas and Taylor 1992)
	NS24UCB	R	20	AAACCTTGTTACGACTTTTA		
Algal 18S rRNA gene	Euk F	F	21	AACCTGGTTGATCCTGCCAGT	1.8 kbp	(Medlin et al. 1988)
	Al1700r^*^	R	18	CTCCTTCCTCTAGGTGGG		(Helms et al. 2001)
V3-V4 region of 16S rRNA gene	341F	F	17	CCTACGGGNGGCWGCAG	460 bp	(Klindworth et al. 2013)
	805R	R	21	GACTACHVGGGTATCTAATCC		(Yergeau et al. 2015)

*Reverse-complement of Al1700f (Helms et al. 2001)

### Amplification and sequencing of V3-V4 region of bacterial 16S rRNA gene

The V3-V4 region of the 16S rRNA gene was amplified using the specific primers 341F and 806R (Table 2). The PCR protocol for the V3-V4 region was initiated by an initial denaturation phase, 30 cycles involving denaturation/annealing/extension, and a final extension step at 95 °C for 3 min, 95 °C for 30 s/55 °C for 30 s/72 °C for 30 s, and 72°C for 5 min. High-quality amplicons were selected for paired-end 300-bp sequencing using the Illumina MiSeq platform (San Diego, CA, USA). Sequencing was performed by Molecular Diagnostic Companies, Environmental Research and Solutions Co. Ltd. (Kyoto, Japan) and SolGent Co. Ltd. (Daejeon, Korea) (Faluaburu et al. 2019; He et al. 2022).

### Sequence data analysis and OTU determination

Sequences obtained from the Sanger method for 18S rRNA genes were rigorously examined using the BioEdit biological sequence alignment editor with the ClustalW program to remove low-quality results (Thompson et al. 1994; Hall 1999). High-quality sequences were retained, assembled manually, and scrutinized by tree topology analysis to check for potential chimeras (Jumpponen 2007). The identification of lichen-forming fungal and algal partners was facilitated using the Basic Local Alignment Search Tool (BLAST) (provided by National Center for Biotechnology Information).

The reads of the V3-V4 region generated using the MiSeq method were uploaded to the EzBioCloud Microbiome Taxonomic Profiling pipeline for processing and analysis (https://www.ezbiocloud.net/contents/16smtp; accessed 05 January 2024) (Yoon et al. 2017). Any reads with lengths < 100 bp and ambiguous reads with low average quality scores (less than 25) were detected and discarded (He et al. 2022). Qualified valid reads were rarefied to approximately 12,000. After dereplicating identical sequences, these deduplicated sequences were subjected to taxonomic assignment using the EzBioCloud prokaryotic sequence database of the 16S rRNA gene, PKSSU4.0. Clear taxonomic rank boundaries were set based on 16S rRNA gene sequence identity cut-off values as follows: 97%, 94.5%, 86.5%, 82.0%, 78.5%, and 75.0% for species, genus, family, order, class and phylum, respectively (Yarza et al. 2014); unclassified reads of species or higher-rank below these cut-off values were appended with the suffix “uc” tentatively. All reads unidentified at the species-like rank (< 97% similarity) were chimera-checked with the EzBioCloud chimera sequence detection algorithms with the reference “16S” database (https://help.ezbiocloud.net/mtp-pipeline/; accessed 05 January 2024); any read identified as a chimera through this analysis was subsequently removed. Prokaryotic singleton and eukaryotic plastid reads were discarded. Remaining V3-V4 reads were clustered into OTUs at a cutoff value of 97% identity (Yoon et al. 2017), and the representative OTUs were searched using BLAST.

Sanger sequences of the fungal 18S rRNA gene were aligned using the ClustalW algorithm included in MEGA-X and then adjusted manually (Thompson et al. 1994; Kumar et al. 2018). A phylogenetic tree was constructed using the maximum-likelihood (ML), maximum-parsimony (MP), and neighbor-joining (NJ) methods (provided by MEGA-X), and the reliability of the inferred tree was tested using 1,000 bootstrap replications.

The sequences derived from the Sanger method for 18S rRNA genes of fungal and algal partners were duly archived in the DNA Data Bank of Japan (DDBJ)/European Nucleotide Archive/GenBank database, and the assigned accession numbers are as LC742247-LC742264 and LC742265-LC742282 for lichen-forming fungi and algae, respectively. The V3-V4 reads are available at the DDBJ Sequence Read Archive (DRA) under the accession number DRA015127. The associated BioProject and BioSample numbers are PRJDB14688 and SAMD00553187–SAMD00553204, respectively. The samples and accession numbers are correspondingly recorded in “Online Resource 1: Table S1–S3”. Details of the V3-V4 read counts and taxonomic classifications are shown in Table 3.

**Table 3:** Counts of OTUs based on 97% similarity, MiSeq-generated V3-V4 region reads, and OTU-derived species (species-like taxa), genera, families, orders, classes, and phyla in each sample

Sample	Picked	Rarefied	OTU	Species	Genus	Family	Order	Class	Phylum	Mean length (bp)
	Read	Valid read								
N01	11,446	11,243	191	165	100	62	39	24	13	401.5
N02	13,042	12,656	336	290	146	66	37	24	13	398.9
N03	12,896	11,286	125	87	44	28	25	17	10	382.4
N04	12,643	12,286	165	126	57	29	23	16	9	398.6
N05	13,161	11,768	143	118	59	34	29	20	11	400.1
N06	11,925	11,476	102	81	42	26	23	17	9	401.5
N07	25,797	11,668	76	65	31	22	21	17	11	403.4
N08	24,179	12,005	163	121	57	30	23	16	9	391.8
N09	11,508	11,334	145	125	68	40	29	19	9	397.3
Sub-total	136,597	105,722	711	518	249	118	67	42	17	397.3
N10	14,186	11,708	490	324	165	75	47	33	16	409.9
N11	14,294	12,235	330	202	95	41	30	18	12	410.1
N12	14,530	12,889	335	167	73	34	28	22	10	403.2
N13	14,421	11,160	260	142	70	35	25	18	11	407.1
N14	19,142	12,671	258	121	41	18	16	12	9	402.6
N15	17,437	12,771	283	163	64	26	23	17	11	402.4
N16	17,303	12,271	120	58	27	12	10	6	5	402.5
N17	14,415	11,191	309	203	104	54	34	23	11	407.3
N18	14,182	12,428	276	171	87	45	31	22	14	412.6
Sub-total	139,910	109,324	1,046	550	252	106	63	40	16	406.4
Total	276,507	215,046	1,395	809	361	155	82	49	19	401.8

Due to sample overlaps, the sub-total and total numbers of taxa are lower than the simple sums. The average read lengths are also provided. Samples N01 to N09 and N10 to N18 were collected from the four regions of the Arctic zone and the subarctic zone, respectively

### Diversity indices and bioinformatic analyses of OTUs

The rarefaction curves were analyzed using the EzBioCloud Microbiome Taxonomic Profiling pipeline. Additionally, to assess the richness and evenness of the bacterial OTUs in the lichen samples, rarefaction curve analysis and computation of alpha-diversity indices encompassing Chao1 richness, Shannon indices, and Simpson indices were performed. It is important to note that the Chao1 index incorporates singleton OTUs into its calculations.

Beta-diversity was assessed using principal component analysis (PCA) and hierarchical cluster analysis (HCA) based on the UniFrac distance matrix, as well as non-metric multidimensional scaling (NMDS) for Arctic and Subarctic OTUs (Kruskal 1964; Lozupone et al. 2007). Linear discriminant analysis (LDA) (Fisher 1936) and the LDA-Effect Size method (LEfSe; http://huttenhower.sph.harvard.edu/galaxy/; accessed 05 January 2024) (Segata et al. 2011), indicator OTUs that distinguish the lichen OTU populations were identified. The threshold LDA score was set to four (He et al. 2022). Differential abundance analysis was performed by analyzing the compositions of the microbiomes with bias correction (ANCOM-BC) (Lin and Peddada 2020), and differential abundance analysis was carried out.

Using the Visualization and Analysis of Networks containing Experimental Data (VANTED) (https://www.cls.uni-konstanz.de/software/vanted/; accessed 05 January 2024) (Junker et al. 2006) and the Phylogenetic Investigation of Communities by Reconstruction of Unobserved States 2.0 (PICRUSt 2.0) programs (Douglas et al. 2020), to mapped OTUs onto established metabolic pathways identified in the Kyoto Encyclopedia of Genes and Genomes (KEGG; http://www.genome.jp/kegg/; accessed 05 January 2024) (Kanehisa et al. 2014).

## Results

### Identification of the fungal and algal partners of the rock-dwelling lichens

The fungal members of the lichens examined in the Canadian Arctic, Norway, Finland, and Canadian Subarctic regions were the closest relatives to the ascomycete *Umbilicaria*, according to a BLAST search of near-full-length 18S rRNA gene sequences. The similarity values were at least 99.07% (Online Resource 1: Table S2). In Canadian Subarctic samples, intraspecific differences in gene sequences were observed.

With similarity values of 97.99% or greater in four regions, the derived algal sequences were most closely linked to the green algal genus *Trebouxia*, which was the known photobiont in the genus *Umbilicaria*, as well as the most prevalent photobiont among lichens (Peršoh et al. 2004; Friedl and Rokitta 1997). (Online Resource 1: Table S3).

## Evaluation of MiSeq-generated V3-V4 Sequences and the derived OTUs

In total, 276,507 MiSeq-derived raw reads from 18 lichen samples were randomly selected. Each sequence was rarefied to approximately 12,000 reads, yielding a total of 215,046 reads. Based on data from the EzBioCloud database (Jumpponen 2007), the mean length of all valid readings for the Arctic and Subarctic was 397.3 bp and 406.4 bp, respectively. Table 3 displays the number of OTUs and the derived species (species-like taxa), genera, families, orders, classes, and phyla obtained from the OTUs in each sample using rarefied reads.

The zonal distributions of the numbers of OTUs, species (species-like taxa), genera, families, orders, classes, and phyla are listed in Table 4, which shows the counts in a Venn diagram that is only in the Arctic, only in the Subarctic, and in both zones. More than half of the order, class, and phylum counts were common in both zones, demonstrating bizonal similarity in higher-rank taxa and zonal uniqueness in lower-rank taxa. Higher frequencies (percentages) of zone-specific OTUs were observed in the ranking of OTUs and species (species-like taxa).

**Table 4:** Distributions of operational taxonomic units, derived species (species-like taxa), genera, families, orders, classes, and phyla found only in Arctic, only in Subarctic, and in both zones. The overall figures match those in Table 3

Distribution	Observed OTU	Species	Genus	Family	Order	Class	Phylum
Only in the arctic zone	349	259	109	49	19	9	3
Only in the subarctic zone	684	291	112	37	15	7	2
Common to both zones	362	259	140	69	48	33	14
Total	1,395	809	361	155	82	49	19

To eliminate the potential impact of excessively different reads on the results, rarefied reads and OTU counts were used to construct rarefaction curves (Online Resource 1: Fig. S1). The coverage, or the ratio of the observed OTU counts to theoretically estimated OTU counts (comparable to Chao1, an alpha-diversity index), ranged from 86.83% in N09 to 99.17% in N16, with an overall average of 93.77% (respective coverages can be calculated from Table 5). As a result, it was believed that there were enough reads produced by MiSeq to perform additional statistical and bioinformatics studies.

**Table 5:** Nine lichen samples from the Arctic zone (N01 to N09) and nine samples from the Subarctic zone (N10 to N18) were used to calculate the alpha-diversity indices such as Chao1, Shannon, and Simpson indices for the observed bacterial OTUs

Sample	Observed OTU	Chao1	Shannon (*ENS*)		Simpson (*ENS*)	
N01	191	200.2	3.68	*39.6*	0.07	*14.3*
N02	336	358.9	4.50	*90.0*	0.03	*33.3*
N03	125	133.3	2.66	*14.3*	0.12	*8.3*
N04	165	178.6	3.36	*28.8*	0.07	*14.3*
N05	143	156.3	3.55	*34.8*	0.05	*20.0*
N06	102	103.7	3.09	*22.0*	0.09	*11.1*
N07	76	83.5	2.37	*10.7*	0.15	*6.7*
N08	163	179.2	3.30	*27.1*	0.06	*16.7*
N09	145	166.8	2.95	*19.1*	0.11	*9.1*
Average	**160.7**	**173.4**	**3.27**	***31.8***	**0.08**	***14.9***
N10	490	558.4	4.09	*59.7*	0.04	*25.0*
N11	330	343.0	3.77	*43.4*	0.05	*20.0*
N12	335	346.5	3.87	*47.9*	0.05	*20.0*
N13	260	272.2	3.54	*34.5*	0.07	*14.3*
N14	258	265.1	3.36	*28.8*	0.07	*14.3*
N15	283	291.8	3.49	*32.8*	0.08	*12.5*
N16	120	121.2	1.74	*5.7*	0.45	*2.2*
N17	309	340.7	3.68	*39.6*	0.06	*16.7*
N18	276	293.2	3.33	*27.9*	0.08	*12.5*
Average	**295.7**	**314.7**	**3.43**	***35.6***	**0.11**	***15.3***

The *ENS* values were calculated from the Shannon and Simpson indices

### Taxonomic composition of lichen-associated bacterial community

**Fig. 2:**
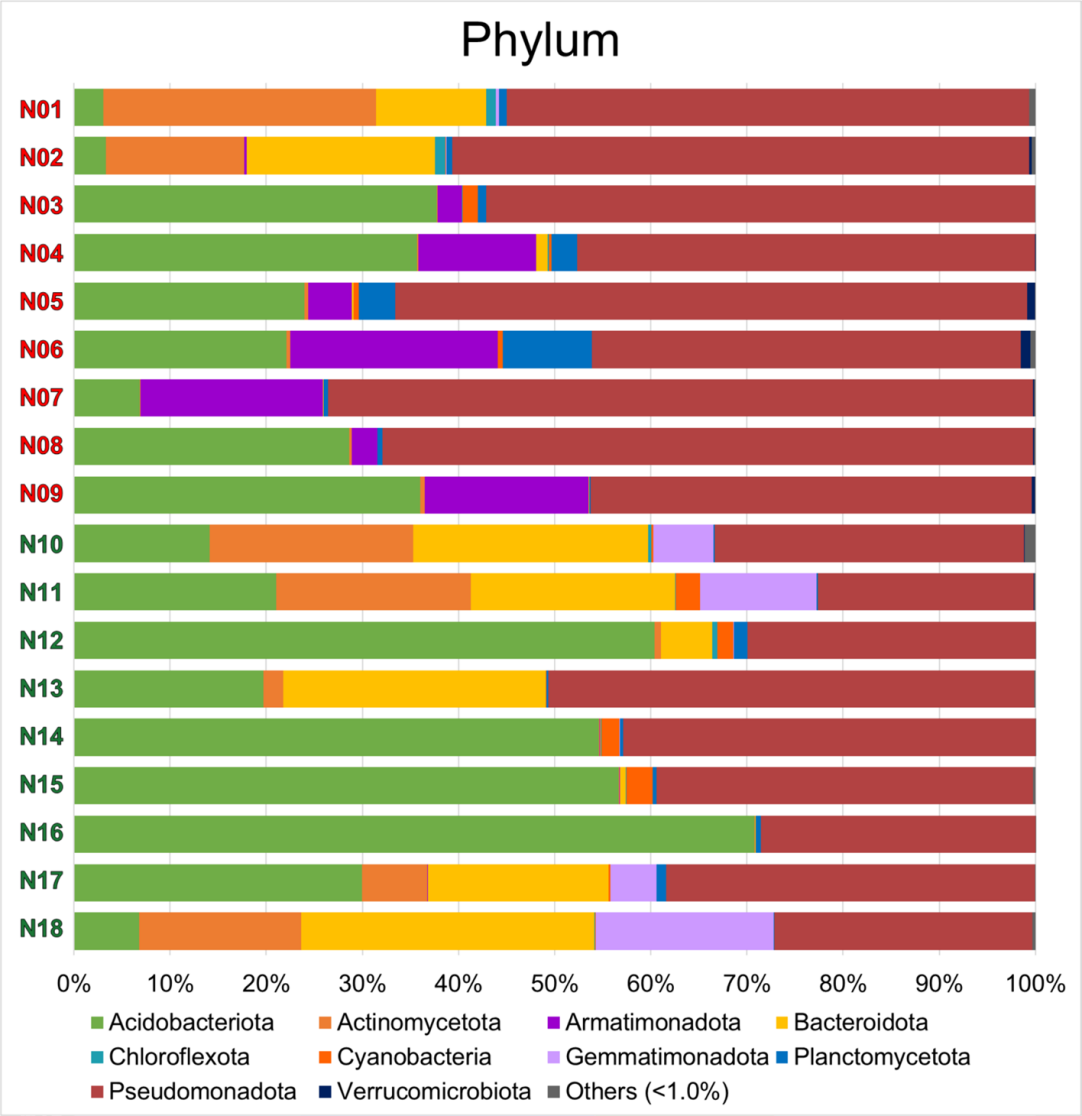
Bacterial phylum compositions of operational taxonomic units from Arctic zone (N01 to N09) and Subarctic zone (N10 to N18) lichen samples. Ten phyla were found to have read abundances greater than 1% of the total reads. “Online Resource 1: Fig. S2–S5” displayed the makeups of the bacterial classes, orders, families, and genera

Figure 2 shows the OTU-derived bacterial phylum composition of the 18 lichen samples using rarefied reads. Ten phyla were shared by all samples based on reads whose abundances were > 1% of the total read counts. A total of 5–16 bacterial phyla were found in each lichen sample (Table 3), with an extra 3–14 phyla based on reads with counts < 1% of the total. Acidobacteriota, Actinomycota (formerly Actinobacteria), Armatimonadota, Bacterroidota (formerly Bacteroidetes), Chloroflexota, Cyanobacteria, Gemmatimonadota, Planctomycetota, Pseudomonadota (formerly Proteobacteria), and Verrucomicrobiota were the most common phyla.

### Alpha- and Beta-diversity

Alpha-diversity indices were generated for each lichen sample to assess the OTU richness (Table 5). In this study, the estimated OTU counts for the rarefaction analysis were based on the Chao1 index values according to previous studies because Chao1 may more accurately represent the diversity of bacterial species in a large sample size (He et al. 2022; Kers and Saccenti 2022; Liu et al. 2022). The effective number of species (*ENS*) was determined using the Shannon and Simpson index values (Jost 2006). Subarctic samples were found to have higher Chao1, Shannon, and *ENS* values as well as higher OTU numbers, indicating increased species richness. For the Subarctic samples, lower Simpson’s index values indicated greater species diversity and evenness.

The Chao1 values as well as the numbers of observed OTU and derived species (species-like taxa; Table 3) were substantially greater than the *ENS* values derived from the Shannon and Simpson indices. Therefore, it was preferable to use Chao1 values to approximate the estimated OTU numbers in this study, and previous studies have confirmed the feasibility of using Chao1 to approximate the estimated OTU numbers (He et al. 2022; Kers and Saccenti 2022; Liu et al. 2022).

**Fig. 3:**
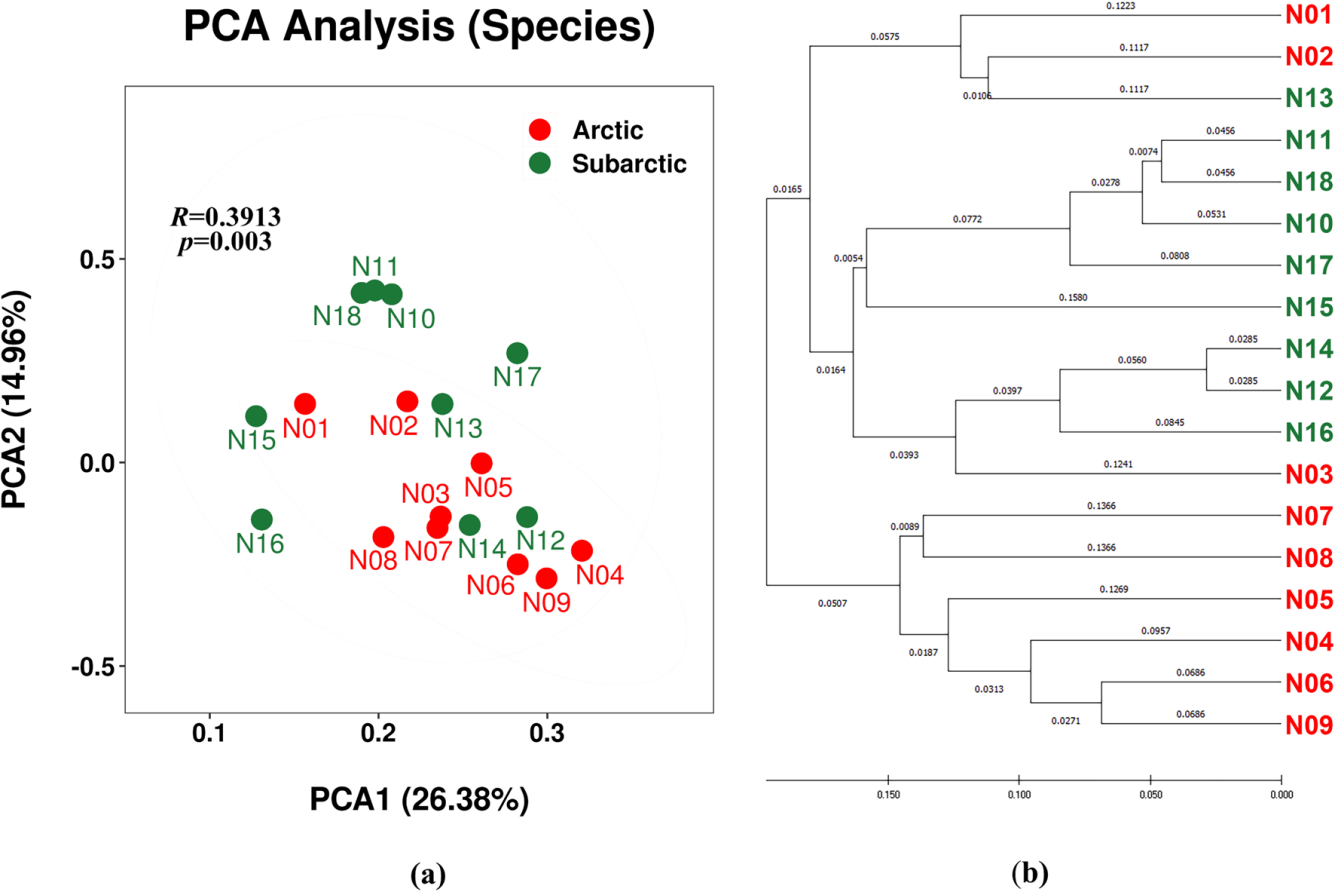
Principal component analysis (PCA) plot (**a**) and hierarchical cluster analysis (HCA) dendrogram (**b**) of the operational taxonomic unit (OTU)-derived bacterial species (species-like taxa) of the lichens collected in the Arctic (red) and Subarctic (green) zones. “Online Resource 1: Fig. S6” displays the PCA plots for the ranks of genus, family, order, class, and phylum

Beta-diversity was evaluated to show sample similarities or dissimilarities using PCA and HCA (Fig. 3). Although the Subarctic zone had higher biodiversity and species richness based on alpha-diversity, PCA significantly revealed that the main species (species-like taxa) of the bacterial communities (the principal components representing data characteristics) were not notable different (*p* < 0.005, |R|< 0.4), as shown in the PCA plot (Fig. 3a). A zonal tendency was observed in the HCA dendrogram (Fig. 3b); however, this tendency was not fully discernible because of partial nesting in the dendrogram. Similar circumstances were also observed in the ranks of the genus, family, order, class, and phylum (Online Resource 1: Fig. S6).

**Fig. 4:**
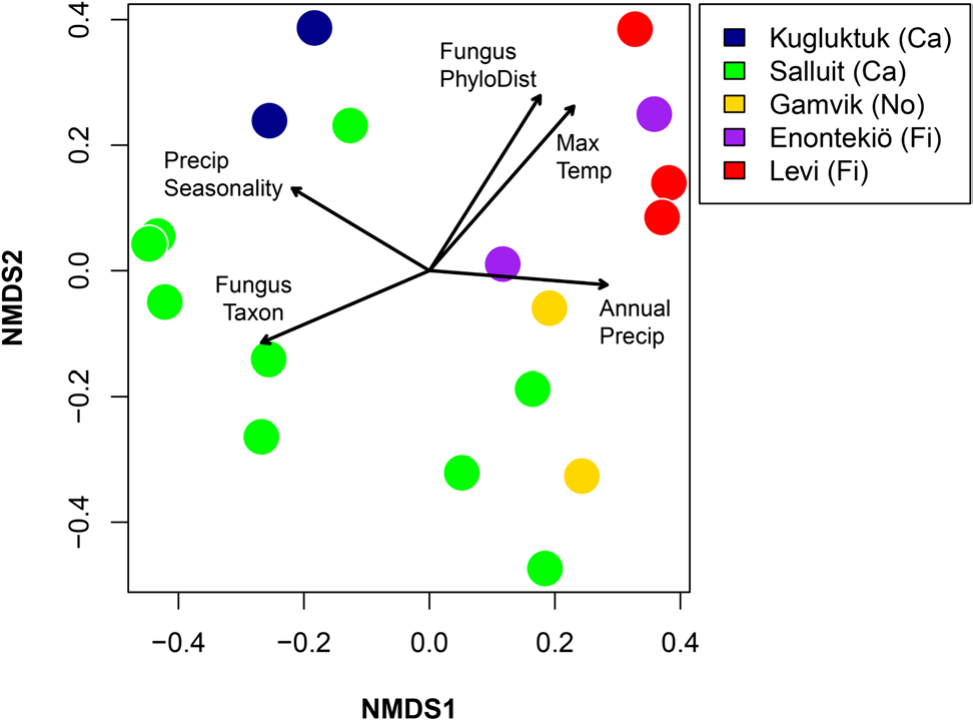
Non-metric multidimensional scaling (NMDS) plot based on Bray–Curtis dissimilarities for the samples from five sampling areas comprising Kugluktuk (Canadian Arctic, blue), Salluit (Canadian Subarctic, green), Gamvik (Norway, yellow), Enontekiö (Finland, purple) and Levi (Finland, red). Discriminant factors including the climatic variables Max Temperature of Warmest Month, Annual Precipitation, Precipitation Seasonality (Coefficient of Variation), the phylogenetic distance (18S rRNA sequence data) of the fungi hosts to the outgroup taxon, and the taxonomic classification of fungi host taxa (based on phylogenetic clustering)

Based on the results presented in Fig. 3 and “Online Resource 1: Fig. S6”, we believe that samples from the Arctic and Subarctic cannot be simply classified by region according to latitude. To identify a more detailed difference, we introduced discriminant factors other than OTUs and combined the OTUs of each sampling area to conduct NMDS analysis (Fig. 4). Available metadata with additional climatic metadata (bioclimatic variables) were obtained from the WorldClim model (Fick and Hijmans 2017).

**Fig. 5:**
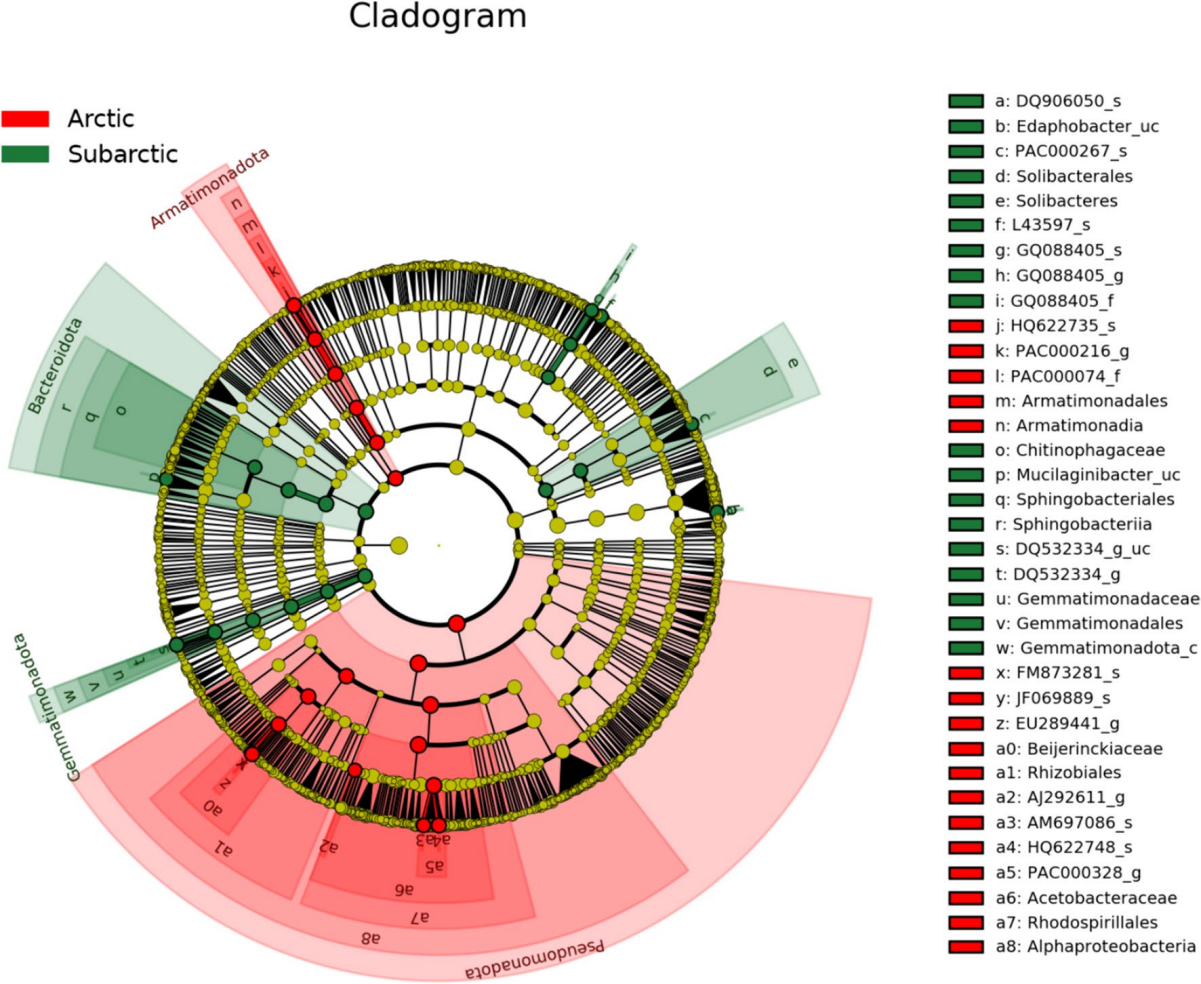
Linear discriminant analysis-effect size (LEfSe) cladogram and Indicator operational taxonomic units (OTUs) in Arctic zone and Subarctic zone. Arctic zone (red) and Subarctic zone (green) lichens were gathered, and the LEfSe cladogram displayed taxonomic indications of bacteria associated with those lichens. Domain Bacteria is represented by the innermost node, which is followed by nodes for each phylum, class, order, family, genus, and species that are organized concentrically. Taxa with significantly higher relative abundance are shown by red and green nodes and tints. Each node circle's diameter relates to the taxon's abundance

Indicator OTUs or taxa, which were screened by LEfSe and depicted in the phylogenetic cladogram, caused a zonal distinction in OTU diversity (Fig. 5). Table 6 lists the significant indications from this group, with LDA scores greater than 4.5. The OTUs of the phylum Pseudomonadota and class Alphaproteobacteria were thus regarded as significant indicators of the Arctic zone, whereas no OTUs from the Subarctic zone showed LDA scores > 4.5, as shown in Table 6.

**Table 6:** Indicator operational taxonomic units (OTUs) and taxa having linear discriminant analysis (LDA) scores > 4.5 identified from the assigned OTU diversity obtained from the Arctic zone

Zone	Code in	Rank of indicator						LDA	*p*-value
	Figure 5	Phylum	Class	Order	Family	Genus	Species	Score	
Arctic	–	Pseudomonadota						5.077	0.0013
	a8	Pseudomonadota	Alphaproteobacteria					5.068	0.00093
	a7	Pseudomonadota	Alphaproteobacteria	Rhodospirillales				4.866	0.0091
	a6	Pseudomonadota	Alphaproteobacteria	Rhodospirillales	Acetobacteraceae			4.863	0.0091
	a5	Pseudomonadota	Alphaproteobacteria	Rhodospirillales	Acetobacteraceae	PAC000328_g		4.794	0.0054
	a1	Pseudomonadota	Alphaproteobacteria	Rhizobiales				4.602	0.0071
	a0	Pseudomonadota	Alphaproteobacteria	Rhizobiales	Beijerinckiaceae			4.571	0.0152
	z	Pseudomonadota	Alphaproteobacteria	Rhizobiales	Beijerinckiaceae	EU289441_g		4.569	0.0119
	–	Armatimonadota						4.537	0.0041
	n	Armatimonadota	Armatimonadia					4.534	0.0031
	m	Armatimonadota	Armatimonadia	Armatimonadales				4.534	0.0031
	l	Armatimonadota	Armatimonadia	Armatimonadales	PAC000074_f			4.534	0.0118
	k	Armatimonadota	Armatimonadia	Armatimonadales	PAC000074_f	PAC000216_g		4.534	0.0118

The Subarctic zone samples were not listed because their LDA scores were less than 4.5

**Fig. 6:**
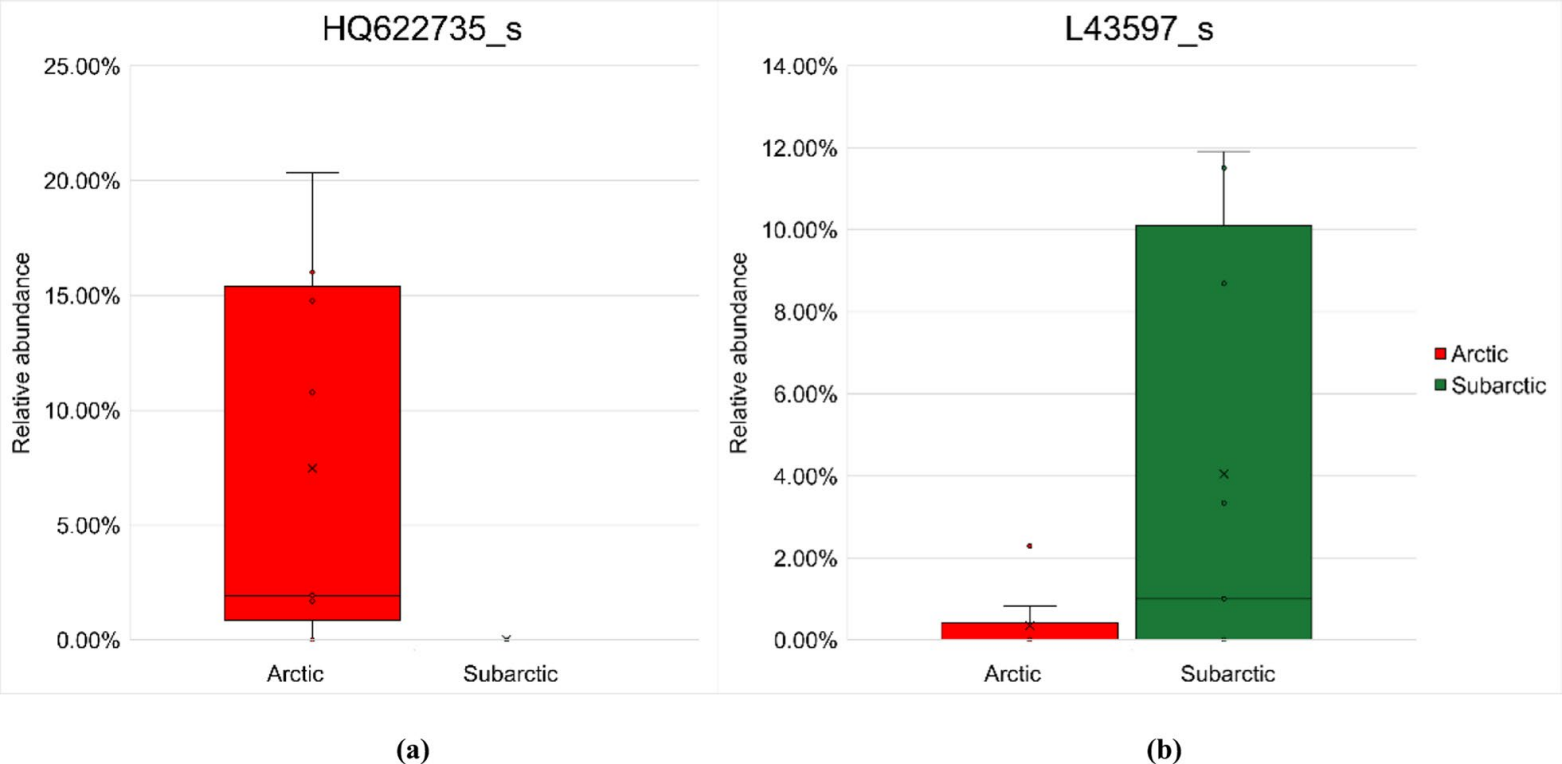
Significant differences (*p* < 0.05) of top indicator operational taxonomic units’ (OTUs’) relative abundances from the Arctic zone (red) and Subarctic zone (green), as determined by analyzing the compositions of the microbiomes with bias correction (ANCOM-BC). **a** the most significant Arctic indicator, HQ622735_s, which belongs to the genus *Capsulimonas* of phylum Armatimonadota. **b** the most significant indicator in the Subarctic, L43597_s indicator, which belongs to the genus *Jatrophihabitans* of phylum Actinomycota. In “Online Resource 1: Fig. S7”, more noteworthy indicator OTUs are displayed

When the LDA score threshold was set to 4, a total of 12 species-level indicator OTUs, five from the Arctic zone and seven from the Subarctic zone, were found and used by ANCOM-BC for differential analysis (Fig. 5). The top indicators from the two zones are shown in Fig. 6 and the other indicators are shown in “Online Resource 1:Fig. S7”.

To forecast the metabolic characteristics of lichen-associated bacteria in both the Arctic and Subarctic zones, PICRUSt defined 12 species-level indicator OTUs with LDA values of > 4. Indicator OTUs were projected onto the KEGG metabolic map, allowing visualization of important metabolic features. Each OTU community displayed five significant pathways at the KEGG Level 1, the highest metabolic category, in descending order of relative abundance: metabolism, genetic information processing, environmental information processing, unclassified, and cellular activities (Online Resource 1: Fig. S8). More than half (50%) of the observed OTUs in the Arctic and Subarctic samples were related to the Level 1 metabolic pathway of “metabolism.”

Overall, the OTUs from both zones had 25 pathways at level 2, which were composed of subcategories (Online Resource 1: Fig. S9). Of these, the top five Level 2 pathways were carbohydrate metabolism (10.30% in Arctic and 11.85% in Subarctic), amino acid metabolism (10.67% in Arctic and 10.71% in Subarctic), membrane transport (10.86% in Arctic and 9.22% in Subarctic), replication and repair (8.11% in Arctic and 8.28% in Subarctic), and energy metabolism (6.69% in Arctic and 5.81% in Subarctic). The “carbohydrate metabolism” pathway was more prevalent in the Subarctic OTUs, however, the “membrane transport” showed the biggest sample-to-sample variation in the metabolic pathways, though the greatest difference was only 1.64%.

**Fig. 7:**
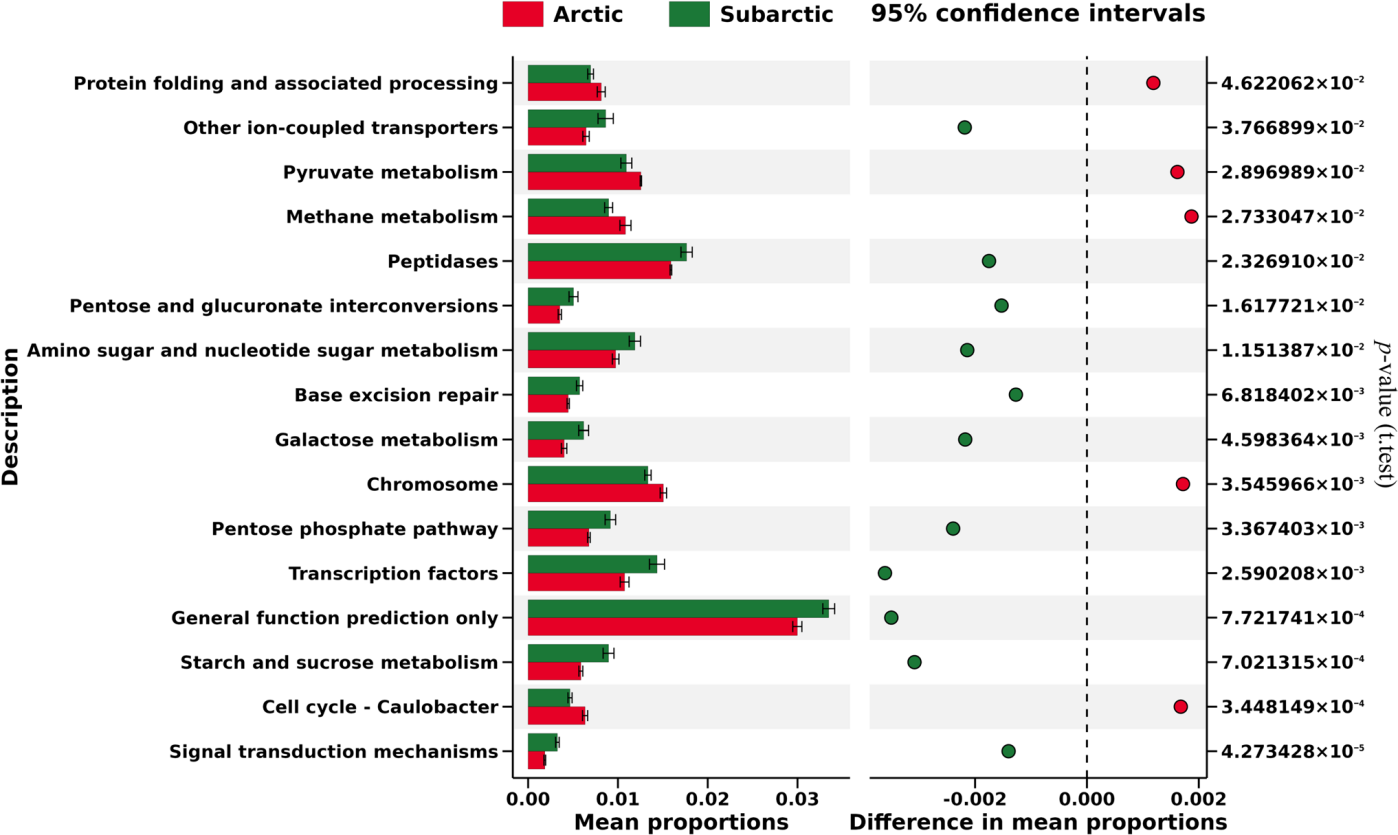
Kyoto Encyclopedia of Genes and Genomes (KEGG) Level 3 metabolic pathway excerpt from the Arctic (red) and Subarctic (green) indicator operational taxonomic units (OTUs). The relative abundance (%) of each pathway to be compared between the two zones is indicated on the horizontal axis. The paths are chosen based on a cutoff value of >|0.0015| for the mean relative abundance distance between two zones. Right side indicators indicate differences that are significant at *p* < 0.05

A total of 191 detailed metabolic pathways at KEGG Level 3 were assigned to all OTUs (Online Resource 1: Fig. S10). The largest sample-to-sample variation was seen for “ABC transporters” (3.07% and 2.36% of total reads in Arctic and in Subarctic, respectively) despite a maximum sample-to-sample difference of only 0.71%, followed by “transporters” (4.90% and 4.36% of total in Arctic and in Subarctic). Moreover, only 16 KEGG Level 3 pathways were chosen from the Arctic (red) and Subarctic (green) indicator OTUs with mean relative abundance distances of two zones at >|0.0015| (significant differences at *p* < 0.05), indicating that the metabolic pathways predicted for the two zones were relatively similar (Fig. 7).

## Discussion

Herbarium specimens as old as 65–114 years yield reliable sequence data (Staats et al. 2011). Therefore, under reasonable methods, the 10-year-old herbarium lichens used in this study (N01 and N02) yielded reasonably acceptable fungal/algal 18S rRNA sequences. The bacterial V3-V4 compositions of the herbarium lichens were not distinctly different from those of freshly collected lichens. Therefore, herbarium specimens and fresh samples were analyzed in this study.

**Fig. 8:**
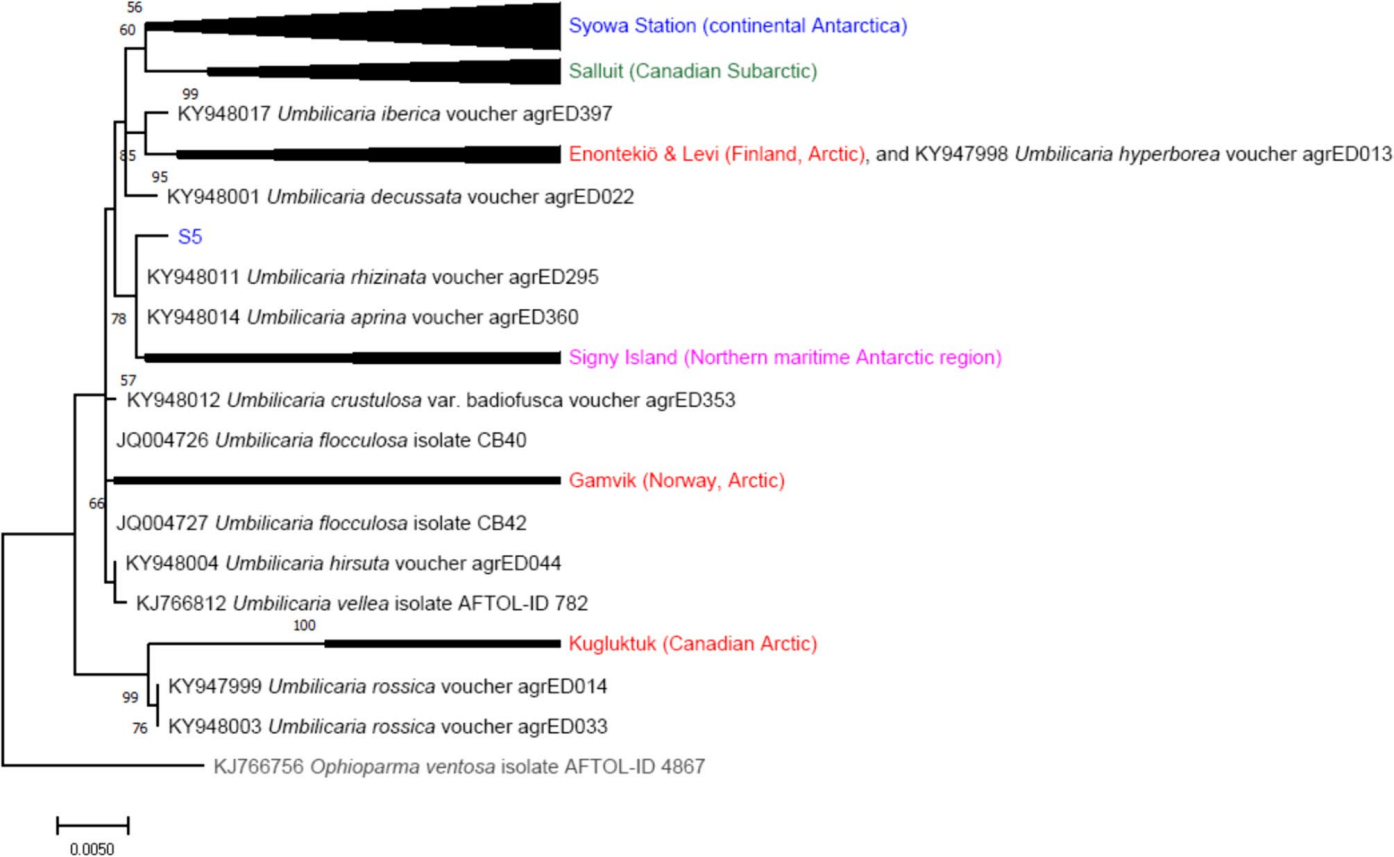
Phylogenetic tree based on 18S rRNA gene sequences of the rock-dwelling *Umbilicaria* lichens collected from Arctic (red) / Subarctic (green) zones (this study), Antarctica zone including Syowa Station (blue) and Signy Island (pink), each closest species (black, italic) by BLAST search and outgroup (gray, italic) (He et al. 2022). The tree was constructed by ML method (provided by MEGA-X). The reliability of the inferred tree was tested by 1,000 bootstrap replications, and the numbers at nodes demonstrate the bootstrap support values (values < 50 not shown). Lengths of the aligned sequences were approximately 1.7 kbp. In addition, in order to increase the reliability, phylogenetic trees were also reconstructed by MP and NJ methods and shown in “Online Resource 1: Fig. S11 & S12”

Rock tripe lichens consisting of fungal partners (mycobionts) of the genus *Umbilicaria* and algal partners (photobionts) of the genus *Trebouxia* were distributed across diverse environments, even including Antarctic ice-free areas which was reported in previous studies (Bates et al. 2011; Faluaburu et al. 2019; Greshake Tzovaras et al. 2020). Variations in 18S rRNA gene sequences were detected for the *Umbilicaria* specimens in this study, namely, *Umbilicaria rossica*, *Umbilicaria freyi*, *Umbilicaria flocculosa*, and *Umbilicaria hyperborea* in the Arctic zone and *Umbilicaria grisea* as reported in the Subarctic zone (Romeike et al. 2002; Faluaburu et al. 2019). We compared the 18S rRNA gene sequences of *Umbilicaria* spp. with those of a previous study conducted in the Antarctic zone, and the results are shown in Fig. 8.

*Umbilicaria*, the fungal partner of lichen samples from the Arctic, Subarctic, Signy Island, and Syowa Station, was more inclined to cluster by region. It is necessary to mention that, except for sample S5 from Syowa Station (continental Antarctica), which is a different *Umbilicaria* sp. and therefore tends to cluster with Signy Island, all other samples belong to the same cluster.

The bacterial OTUs associated with the above *Umbilicaria* in Arctic and Subarctic samples varied in species and genus ranks, as shown in the results of both PCA and HCA (Fig. 3 and “Online Resource 1: Fig. S6”). Thus, the associated bacterial communities largely lacked host-specific grouping/clustering and the results showed no lichen species-specific grouping. Although the lichen samples included various *Umbilicaria* spp., the bacterial OTUs were clustered with partial nesting on the dendrogram between different sampling regions, the Arctic and the Subarctic. Previous studies have shown that regardless of the same *Umbilicaria* sp., bacterial OTUs are clearly clustered by geographical region, at least at the genus level (He et al. 2022; and another submitted manuscript). We believe that the geographical and/or bioclimatic environment and the different lichen-forming fungal species mainly and partially influenced the bacterial microbiomes linked to the *Umbilicaria* spp. investigated here, respectively, and that the climatic features of the sampling sites may actually have similarities.

As another possibility that may have affected the bacterial community structure, we cannot rule out the influence of algal species on lichens. Different species within *Trebouxia* may lead to different bacterial community structures. The algal 18S rRNA gene sequences from the Arctic and Subarctic samples showed 97.6–98.3% and 98.1–99.6% similarity, respectively. In addition, the sequence similarity of the Arctic and Subarctic-derived sequences to each other is different in the range of 96.6–98.8%. Although they are closely related phylogenetically, further research is needed to identify and compare the bacteria that are highly correlated with each species of algae.

Even though the valid reads were rarefied to the same level, the alpha-diversity of the Subarctic samples was still higher than that of the Arctic samples. In terms of the beta-diversity results, including the PCA and HCA on species and genus rank, the Arctic and Subarctic regions were not clearly distinct (Fig. 3 and “Online Resource 1: Fig. S6”). This shows that, although the samples from the two sampling areas have similarities in terms of family, order, class, and phylum rank, the species-like taxa tended to be different. In addition, some samples from the Subarctic showed a clustering trend at different taxonomic ranks, but this was not related to the sampling area (Table 1).

As mentioned in the Results section, we believe that the samples from the Arctic and Subarctic cannot be simply classified by region according to latitude, and we introduced discriminant factors other than OTUs and combined them with the OTUs of each sampling area to conduct NMDS analysis (Fig. 4) to find a more detailed difference. The NMDS results suggested that climate selects host taxa that are associated with different bacterial communities. There is also a direct influence of climate on the bacterial community composition; that is, the bacterial microbiomes from different regions have a very close relationship with the sample location and local climate; however, this study was not able to test both the selection of host taxa by climate and the direct selection of bacterial taxa by climate due to limitations of the dataset.

As mentioned above, the predictions of metabolic pathways based on bacterial microbiome in lichen samples from two zones were similar, even though the highest variation is metabolic pathway "ABC transporters,” the difference was just 0.71%. These somewhat similar cold climates (Sect. Sampling of Rock-dwelling *Umbilicaria* Lichens) may be related to the metabolic pathways linked to the activities anticipated for the OTUs from two different locations. Regardless of acclimatization or evolutionary adaptation, metabolism often adapts to variations in temperature (Clarke and Fraser 2004). Although the bacterial OTUs were not identical, the PICRUSt2.0 anticipated results suggested that similar climates may benefit bacterial OTUs with similar metabolic activities. It should be noted that the presence or absence of reference genomes phylogenetically related to the identified OTUs affects the accuracy of PICRUSt-derived metabolic profiles. It will be necessary to isolate and characterize lichen-dwelling bacteria to specifically analyze and elaborate on their metabolic characteristics, or to reconstruct and analyze bacterial genomes using culture-independent metagenomic approaches. Additionally, the metabolism of other lichen partners, fungi, and algae/cyanobacteria, which also scales with temperature, may have a direct or indirect impact on the associated bacterial microbiomes. Metabolic interactions between bacterial microbiomes and their fungal/algal partners may differ in lichens; therefore, careful selection is required. Through analyses based on OTUs, it is possible to comprehend the ecological and functional roles of bacterial microbiomes as third partners in the lichen symbiosis. However, more information is required to expand this field of study.

## Supplementary Information

Below is the link to the electronic supplementary material.

Supplementary file1 (PDF 2215 kb)

## Declarations

### Data availability

The fungal and algal 18S rRNA gene sequences obtained in this study are deposited in DDBJ under the accession numbers LC742247–LC742264 and LC742265–LC742282, respectively. The MiSeq raw sequence, project, and sample data are available from the DDBJ Sequence Read Archive (DRA015127), BioProject (PRJDB14688), and BioSample (SAMD00553187–SAMD00553204), respectively.
