# Supplementary material for: Bacterial phylotypes associated with rock-dwelling Umbilicaria Lichens from Arctic/Subarctic areas in North America and Northern Europe
Source: Polar Biol. 2024 Oct 21;47(12):1527–41. doi: 10.1007/s00300-024-03303-3 (PMC11604754; doi:10.1007/s00300-024-03303-3)
Supplement: Supplementary file 1 — Supplementary file1 (PDF 2215 kb) [file 300_2024_3303_MOESM1_ESM.pdf]

# Online Resource 1: Supplementary materials (Tables S1 to S3, and Figs. S1 to S10) of "Bacterial Phylotypes Associated with Rock-dwelling *Umbilicaria* Lichens from Arctic/Subarctic Areas in North America and Northern Europe"

**Zichen He**

Graduate School of Integrated Science for Life, Hiroshima University, Higashi-hiroshima 739-8528, Japan

szichenhe@gmail.com

ORCID 0009-0001-9402-1210

**Takeshi Naganuma**

Corresponding author

Graduate School of Integrated Science for Life, Hiroshima University, Higashi-hiroshima 739-8528, Japan

takn@hiroshima-u.ac.jp

ORCID 0000-0003-1925-9461

+81-82-424-7986

**Merry Sailonga Faluaburu**

Graduate School of Biosphere Science, Hiroshima University, Higashi-hiroshima 739-8528, Japan

Current address: Solomon Islands National University, P. O. Box R311, Kukum Highway, Honiara, Solomon Islands

solovalu@gmail.com

**Ryosuke Nakai**

Bioproduction Research Institute, National Institute of Advanced Industrial Science and Technology, Sapporo 062-8517, Japan

nakai-ryosuke@aist.go.jp

ORCID 0000-0002-3078-6695

**Hiroshi Kanda**

National Institute of Polar Research, 10-3 Midori-Cho, Tachikawa 190-8518, Japan

kanda.hiroshi@hotmail.co.jp

**Masaki Uchida**

National Institute of Polar Research, 10-3 Midori-Cho, Tachikawa 190-8518, Japan

uchida@nipr.ac.jp

**Satoshi Imura**

National Institute of Polar Research, 10-3 Midori-Cho, Tachikawa 190-8518, Japan

imura@nipr.ac.jp

ORCID 0000-0002-6803-6996

**Martin W. Hahn**

Research Department for Limnology, Universität Innsbruck, Mondsee A-5310, Austria

martin.hahn@uibk.ac.at

ORCID 0000-0003-0501-2556

**Corresponding author**: Takeshi Naganuma, takn@hiroshima-u.ac.jp, ORCID 0000-0003-1925-9461, +81-82-424-7986

Table S1 BioProject numbers, DRA accession numbers, and BioSample accession numbers associated with the sequence datasets of the V3-V4 region that have been deposited in the public DDBJ database.

| Sample | DRA acc. # | BioProject # | BioSample acc. # |
|---|---|---|---|
| N01 | DRA015127 | PRJDB14688 | SAMD00553187 |
| N02 | | | SAMD00553188 |
| N03 | | | SAMD00553189 |
| N04 | | | SAMD00553190 |
| N05 | | | SAMD00553191 |
| N06 | | | SAMD00553192 |
| N07 | | | SAMD00553193 |
| N08 | | | SAMD00553194 |
| N09 | | | SAMD00553195 |
| N10 | | | SAMD00553196 |
| N11 | | | SAMD00553197 |
| N12 | | | SAMD00553198 |
| N13 | | | SAMD00553199 |
| N14 | | | SAMD00553200 |
| N15 | | | SAMD00553201 |
| N16 | | | SAMD00553202 |
| N17 | | | SAMD00553203 |
| N18 | | | SAMD00553204 |

**Table S2** Accession numbers for sequences of near-full-length fungal 18S rRNA genes originating from the examined rock tripe lichen samples. The table also includes corresponding lengths, the most closely associated species with their respective accession numbers and lengths, and the corresponding similarity values (%).

| Sample | < Accession # | Closest species | < Accession # | Similarity (%) |
|---|---|---|---|---|
| N01 | LC742247 | *Umbilicaria rossica* | KY947999 | 99.74 |
| | | *Umbilicaria rossica* | KY948003 | 99.68 |
| N02 | LC742248 | *Umbilicaria rossica* | KY947999 | 99.81 |
| | | *Umbilicaria rossica* | KY948003 | 99.74 |
| N03 | LC742249 | *Umbilicaria flocculosa* | JQ004726 | 99.73 |
| | | *Umbilicaria flocculosa* | JQ004727 | 99.63 |
| N04 | LC742250 | *Umbilicaria flocculosa* | JQ004726 | 99.74 |
| | | *Umbilicaria flocculosa* | JQ004727 | 99.63 |
| N05 | LC742251 | *Umbilicaria hyperborea* | KY947998 | 99.82 |
| | | *Umbilicaria iberica* | KY948017 | 99.46 |
| N06 | LC742252 | *Umbilicaria hyperborea* | KY947998 | 99.64 |
| | | *Umbilicaria iberica* | KY948017 | 99.29 |
| N07 | LC742253 | *Umbilicaria hyperborea* | KY947998 | 99.70 |
| | | *Umbilicaria iberica* | KY948017 | 99.35 |
| N08 | LC742254 | *Umbilicaria hyperborea* | KY947998 | 99.70 |
| | | *Umbilicaria iberica* | KY948017 | 99.35 |
| N09 | LC742255 | *Umbilicaria hyperborea* | KY947998 | 99.82 |
| | | *Umbilicaria iberica* | KY948017 | 99.46 |
| N10 | LC742256 | *Umbilicaria crustulosa* | KY948012 | 99.16 |
| | | *Umbilicaria hirsuta* | KY948004 | 99.13 |
| N11 | LC742257 | *Umbilicaria crustulosa* | KY948012 | 99.16 |
| | | *Umbilicaria hirsuta* | KY948004 | 99.13 |
| N12 | LC742258 | *Umbilicaria crustulosa* | KY948012 | 99.14 |
| | | *Umbilicaria hirsuta* | KY948004 | 99.12 |
| N13 | LC742259 | *Umbilicaria crustulosa* | KY948012 | 99.21 |
| | | *Umbilicaria hirsuta* | KY948004 | 99.19 |
| N14 | LC742260 | *Umbilicaria hirsuta* | KY948004 | 99.14 |
| | | *Umbilicaria aprina* | KY948014 | 99.12 |
| N15 | LC742261 | *Umbilicaria hirsuta* | KY948004 | 99.14 |
| | | *Umbilicaria aprina* | KY948014 | 99.12 |
| N16 | LC742262 | *Umbilicaria hirsuta* | KY948004 | 99.14 |
| | | *Umbilicaria aprina* | KY948014 | 99.06 |
| N17 | LC742263 | *Umbilicaria hirsuta* | KY948004 | 99.15 |
| | | *Umbilicaria vellea* | KJ766812 | 99.07 |
| N18 | LC742264 | *Umbilicaria crustulosa* | KY948012 | 99.21 |
| | | *Umbilicaria hirsuta* | KY948004 | 99.18 |

**Table S3** Accession numbers for sequences of near-full-length algal 18S rRNA gene originating from the examined rock tripe lichen samples. The table also includes the corresponding lengths, most closely associated species with their respective accession numbers, and corresponding similarity values (%).

| Sample | < Accession # | Closest species | < Accession # | Similarity (%) |
|---|---|---|---|---|
| N01 | LC742265 | *Trebouxia aggregata* | MT901379 | 98.12 |
| N02 | LC742266 | *Trebouxia aggregata* | MT901379 | 97.99 |
| N03 | LC742267 | *Trebouxia jamesii* | Z68700 | 99.34 |
| N04 | LC742268 | *Trebouxia aggregata* | MT901379 | 99.40 |
| N05 | LC742269 | *Trebouxia jamesii* | Z68700 | 99.34 |
| N06 | LC742270 | *Trebouxia jamesii* | Z68700 | 99.34 |
| N07 | LC742271 | *Trebouxia aggregata* | MT901379 | 99.09 |
| N08 | LC742272 | *Trebouxia aggregata* | MT901379 | 99.27 |
| N09 | LC742273 | *Trebouxia jamesii* | Z68700 | 98.21 |
| N10 | LC742274 | *Trebouxia aggregata* | MT901379 | 99.40 |
| N11 | LC742275 | *Trebouxia jamesii* | Z68700 | 99.40 |
| N12 | LC742276 | *Trebouxia aggregata* | MT901379 | 99.40 |
| N13 | LC742277 | *Trebouxia aggregata* | MT901379 | 99.40 |
| N14 | LC742278 | *Trebouxia decolorans* | MK005126 | 98.95 |
| N15 | LC742279 | *Trebouxia jamesii* | Z68700 | 98.43 |
| N16 | LC742280 | *Trebouxia jamesii* | Z68700 | 99.30 |
| N17 | LC742281 | *Trebouxia decolorans* | MK005126 | 99.35 |
| N18 | LC742282 | *Trebouxia aggregata* | MT901379 | 99.27 |

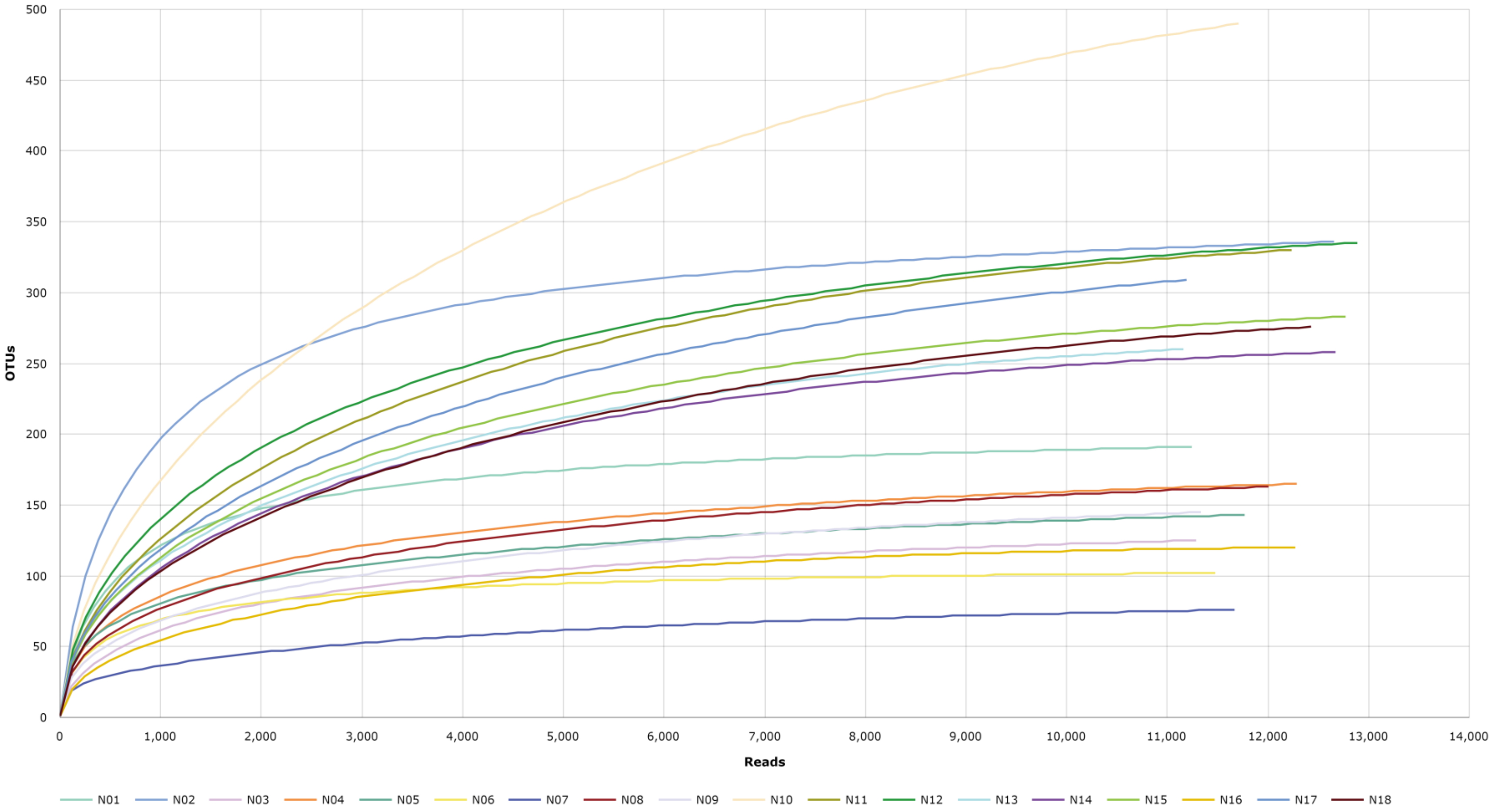


Fig. S1 Illustrated rarefaction curves derived from the read and OTU counts of nine Arctic sites in Kugluktuk (Canada), Enontekiö (Finland), Levi (Finland), and Gamvik (Norway) and nine Subarctic sites in Salluit (Canada).

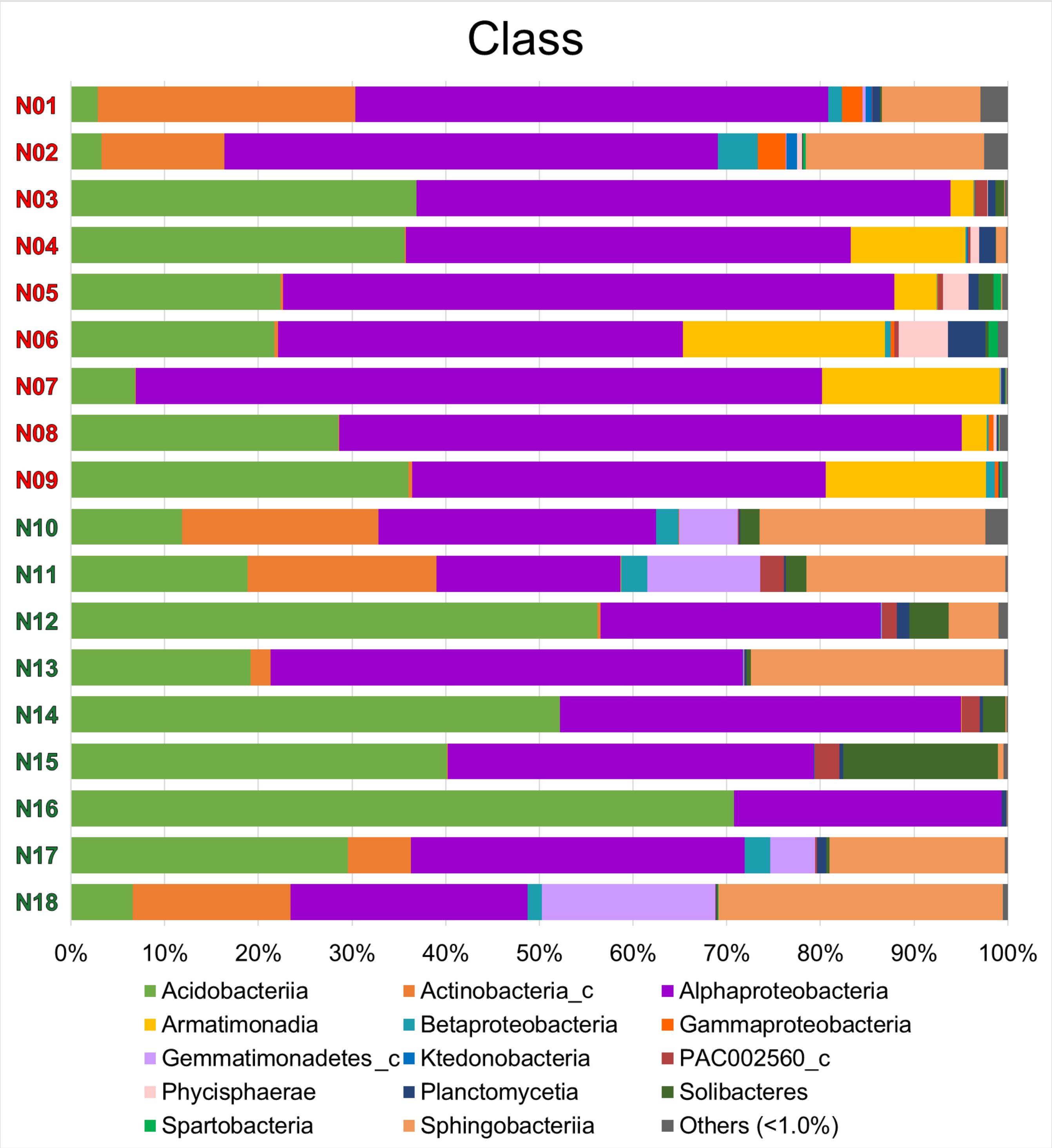


Fig. S2 Bacterial class compositions of OTUs in lichen samples from the Arctic (N01–N09) and Subarctic sites (N10–N18).

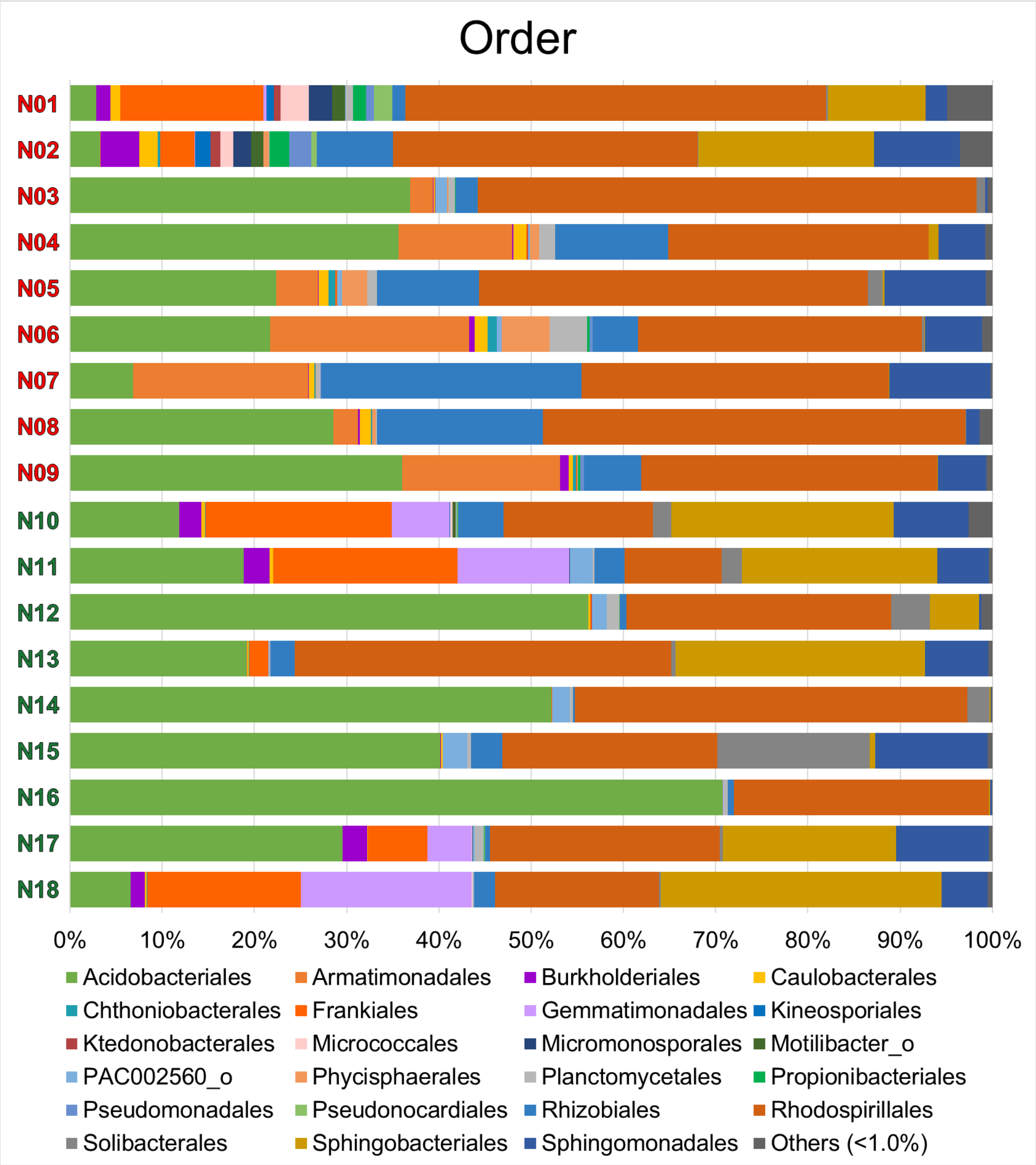


57

Fig. S3 Bacterial order compositions of OTUs in lichen samples from Arctic sites (N01–N09) and 58
Subarctic sites (N10–N18). 59

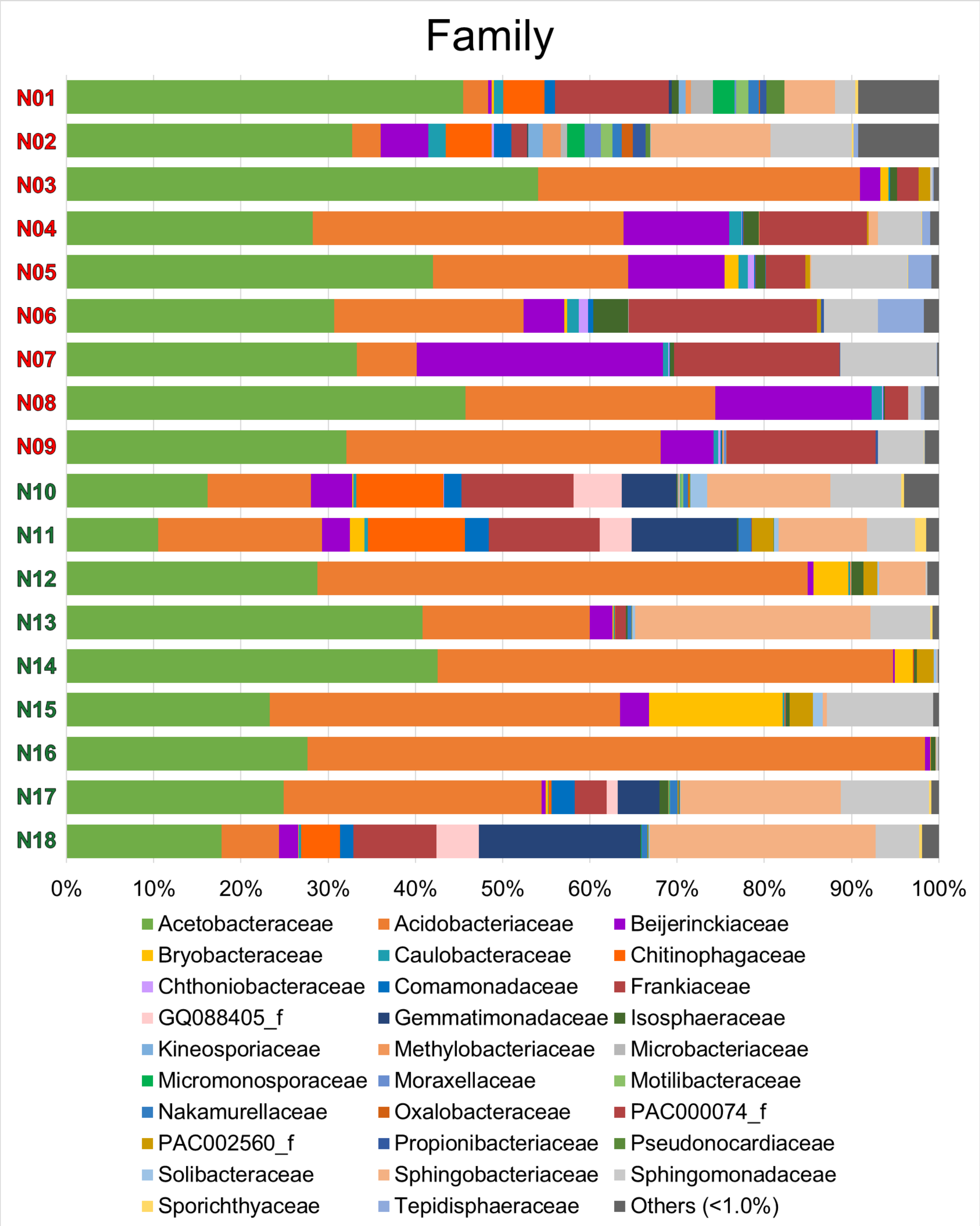


60

Fig. S4 Bacterial family compositions of OTUs in lichen samples from the Arctic (N01–N09) and 61
Subarctic sites (N10–N18). 62

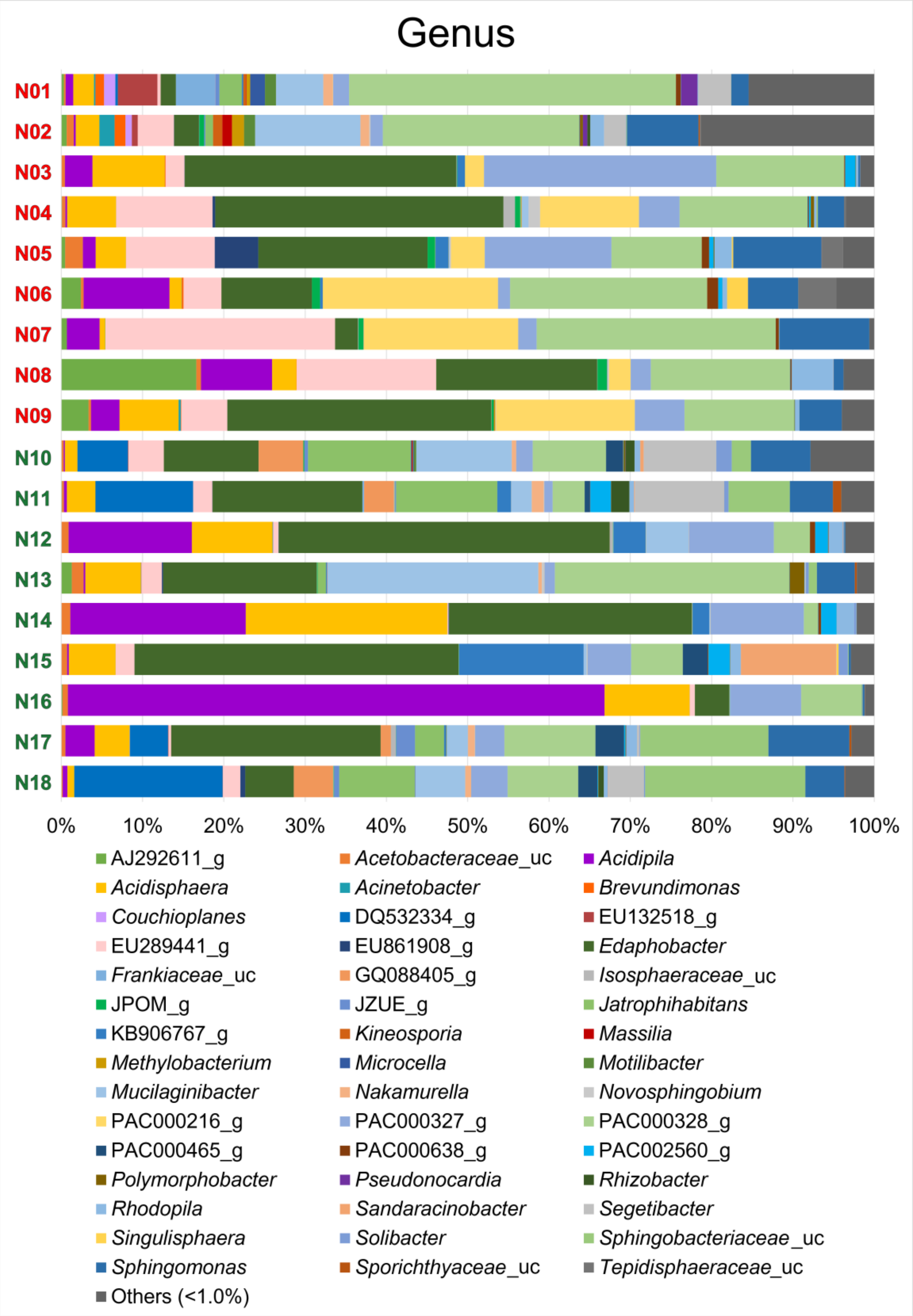


63

Fig. S5 Bacterial genus composition of OTUs in lichen samples from the Arctic (N01–N09) and Subarctic 64
sites (N10–N18). 65

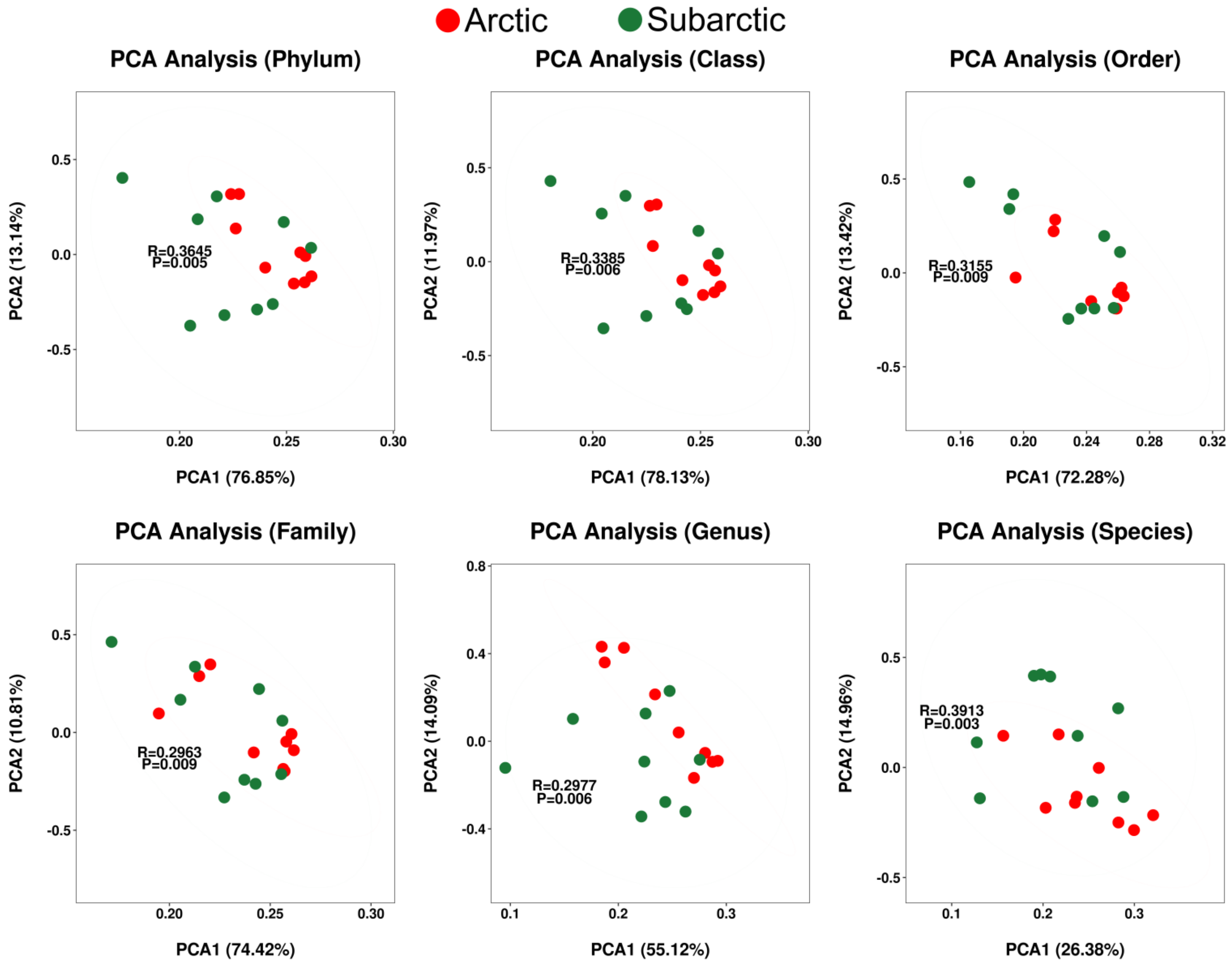


Fig. S6 PCA plots were generated to visualize the distribution of OTU-derived phyla (top-left), classes (top-middle), orders (top-right), families (bottom-left), genera (bottom-middle) and species (bottom-right) among lichen samples collected from the Arctic (red) and Subarctic sites (green).

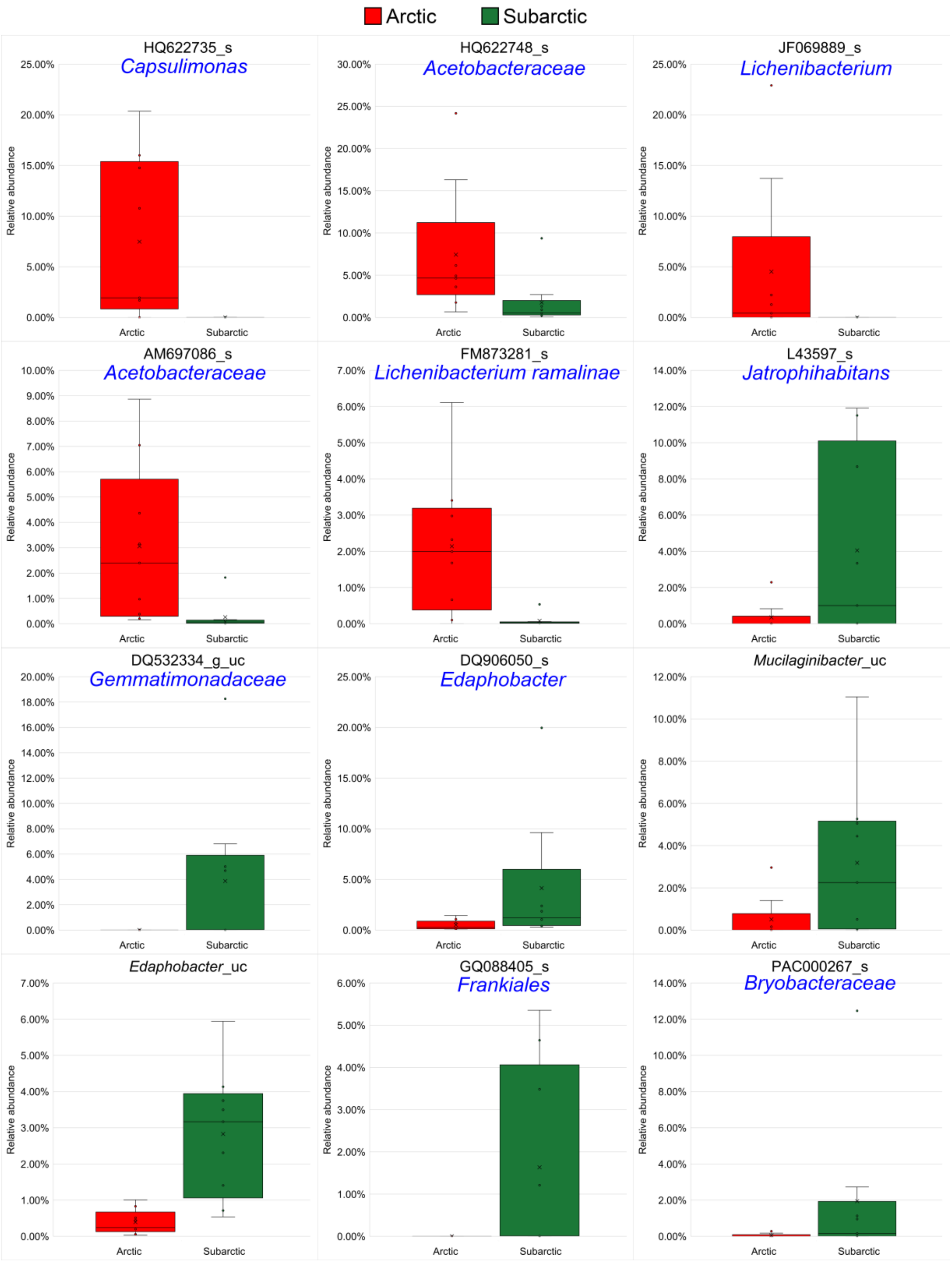


Fig. S7 Statistically significant disparities ($p < 0.05$) in the relative abundances of indicator OTUs were assessed using ANCOM-BC between the Arctic (red) and Subarctic (green) sites. The names of the potentially associated genera or phyla, if applicable, are displayed below their respective OTU identifiers.

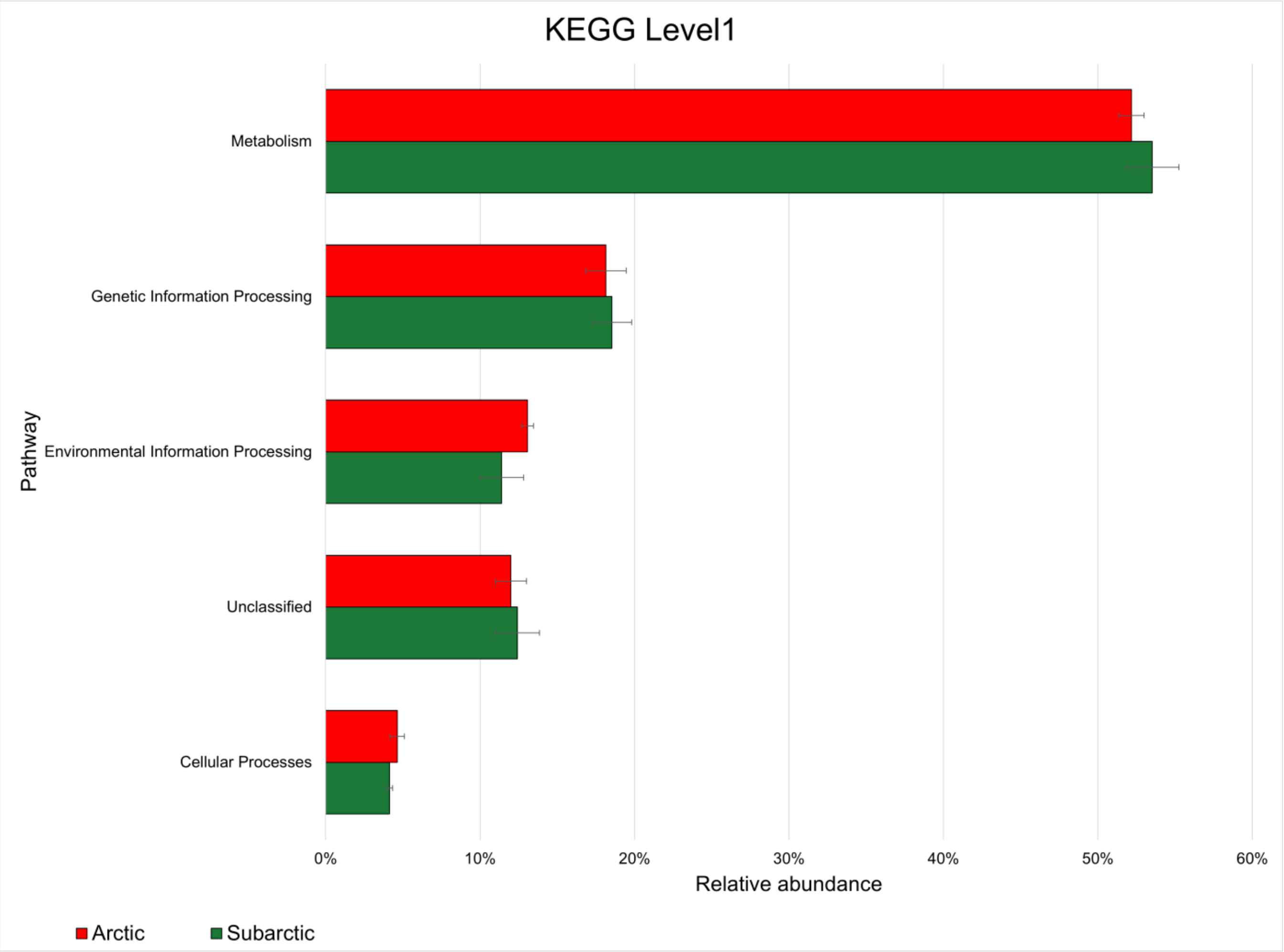


Fig. S8 Metabolic pathways at KEGG Level 1 identified within the indicator OTUs originating from the Arctic (red) and Subarctic sites (green).

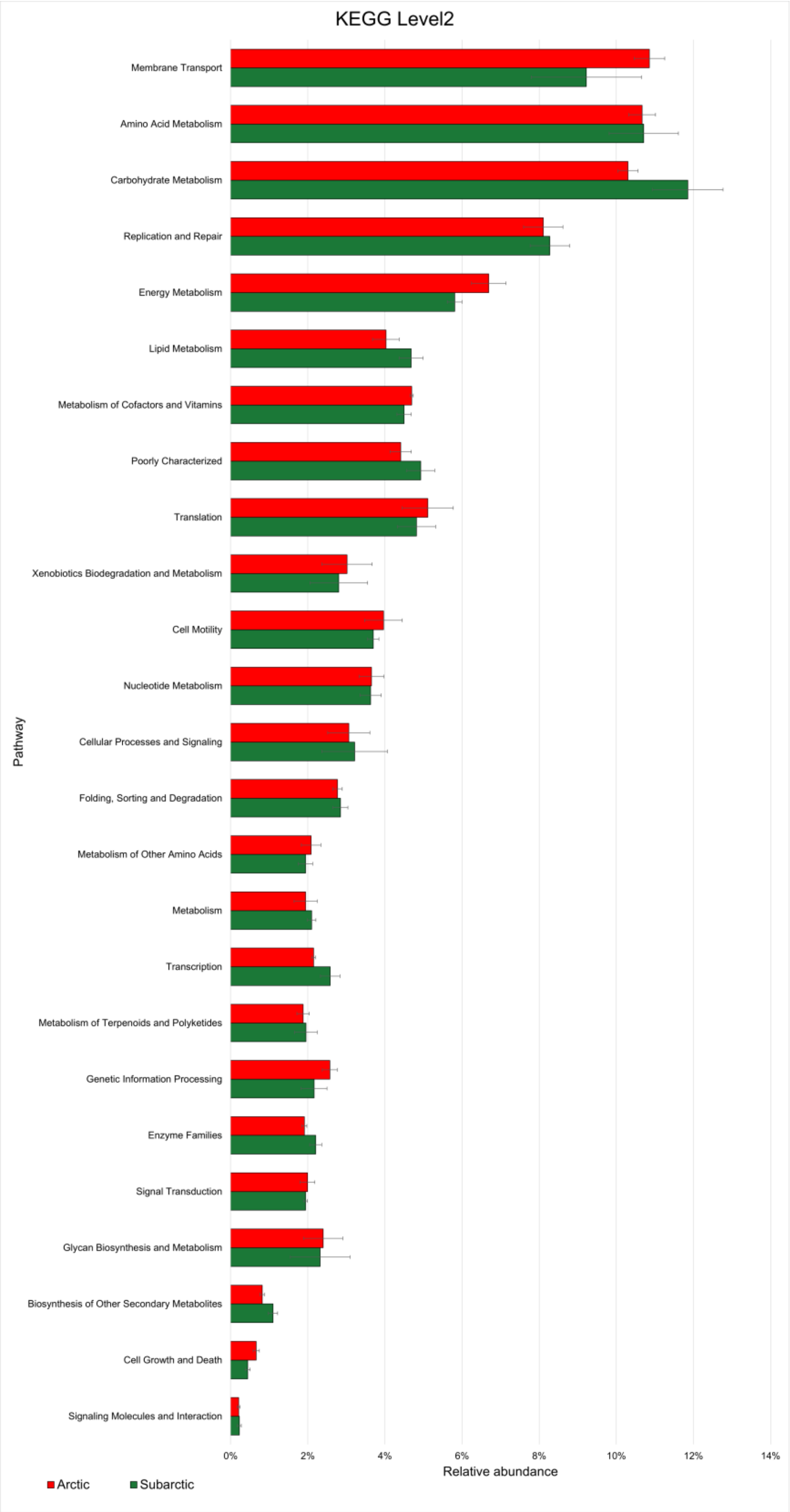


78

Fig. S9 Metabolic pathways at KEGG Level 2 identified within the indicator OTUs originating from the 79
Arctic (red) and Subarctic sites (green). 80

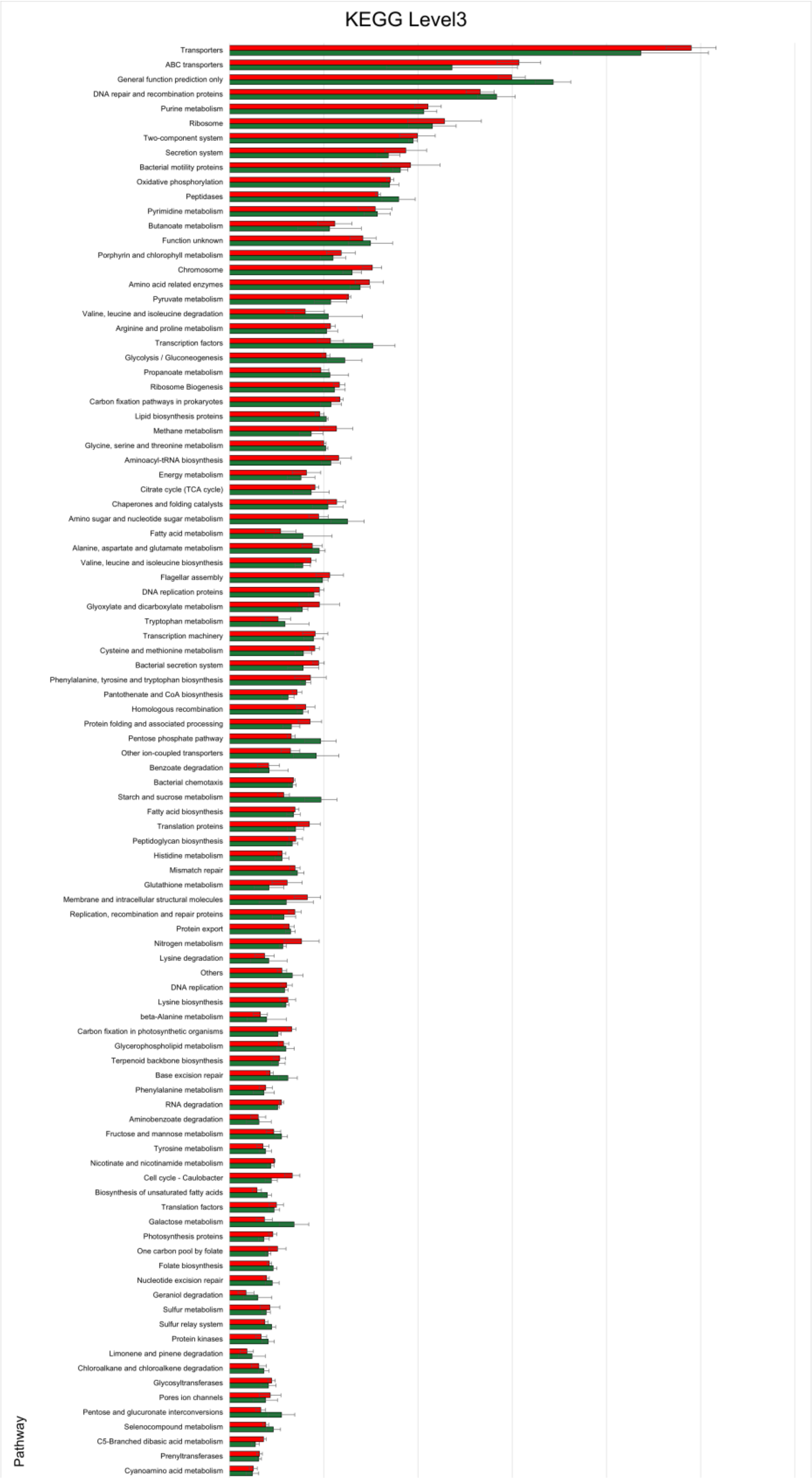


Fig. S10 Metabolic pathways at KEGG Level 3 identified within the indicator OTUs originating from the Arctic (red) and Subarctic sites (green).

(Fig. S10, continued)

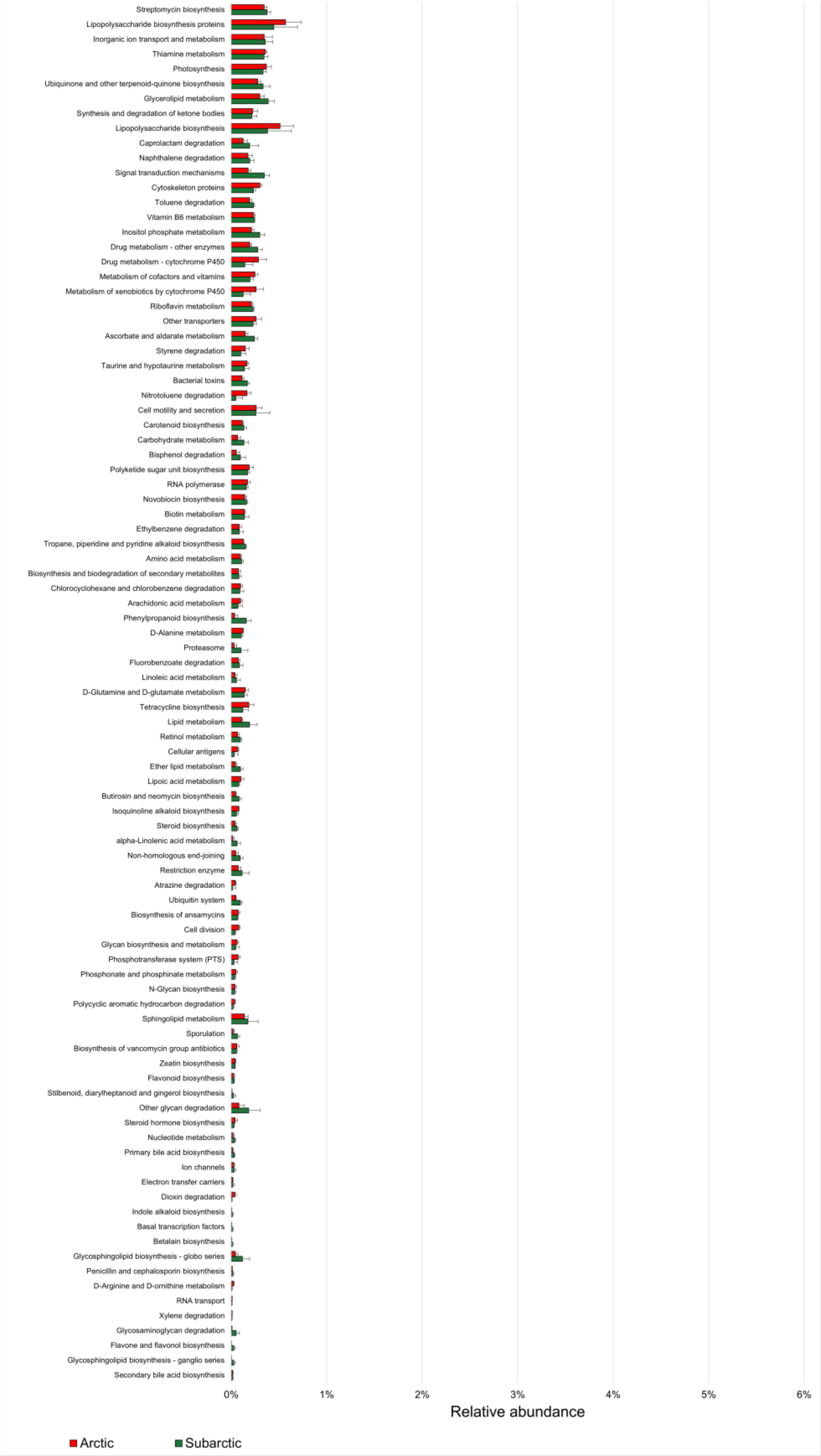

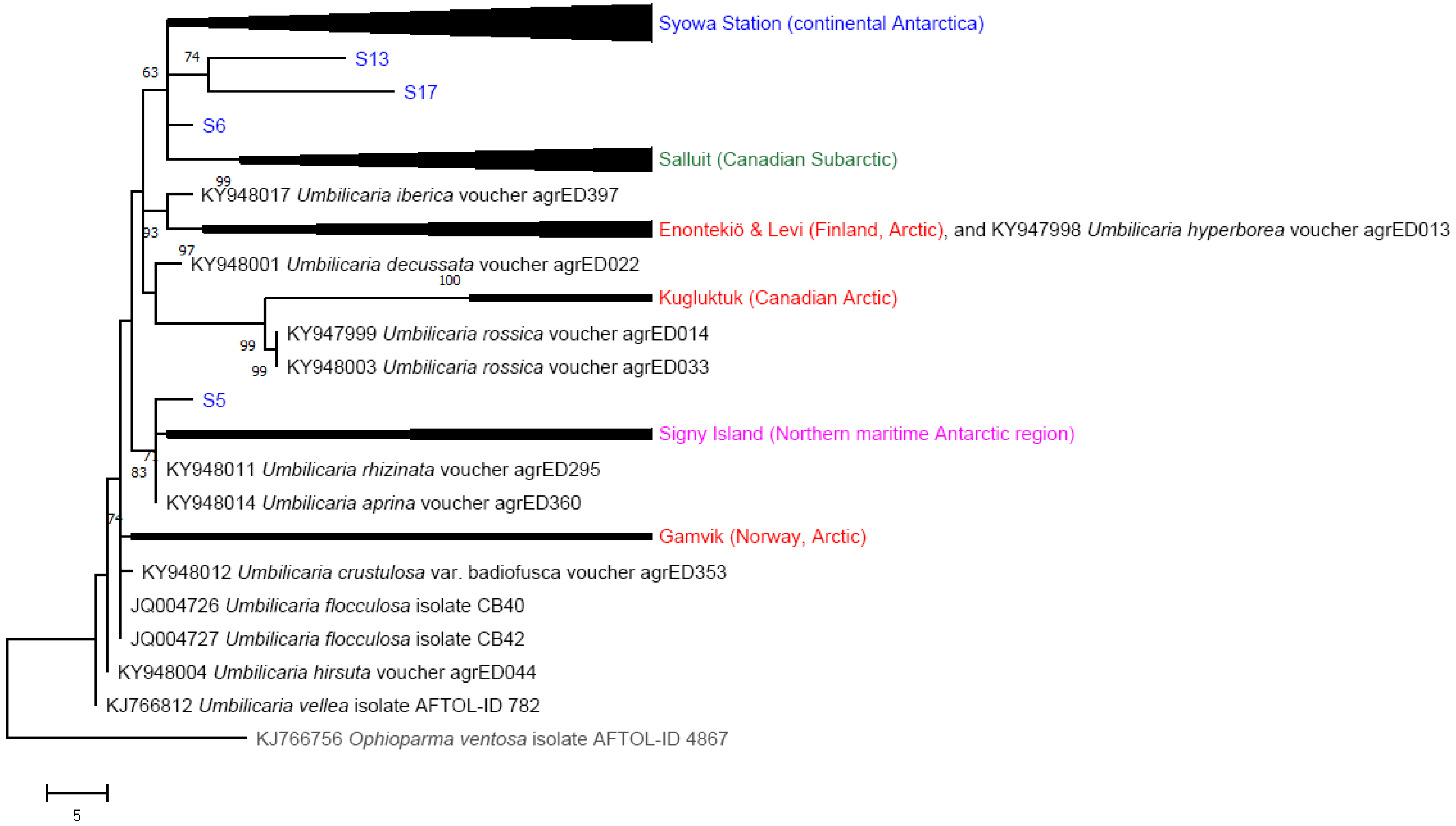


Fig. S11 Phylogenetic tree based on 18S rRNA gene sequences of the rock-dwelling *Umbilicaria* lichens collected from Arctic (red) / Subarctic (green) zones (this study), Antarctica zones including Syowa Station (blue) and Signy Island (pink), each closest species (black, italic) by BLAST search and outgroup (gray, italic) (He et al. 2022). The tree was constructed by Maximum-Parsimony method (provided by MEGA-X). The reliability of the inferred tree was tested by 1000 bootstrap replications, and the numbers at nodes demonstrate the bootstrap support values (values < 50 not shown). Lengths of the aligned sequences were around 1.7 kb.

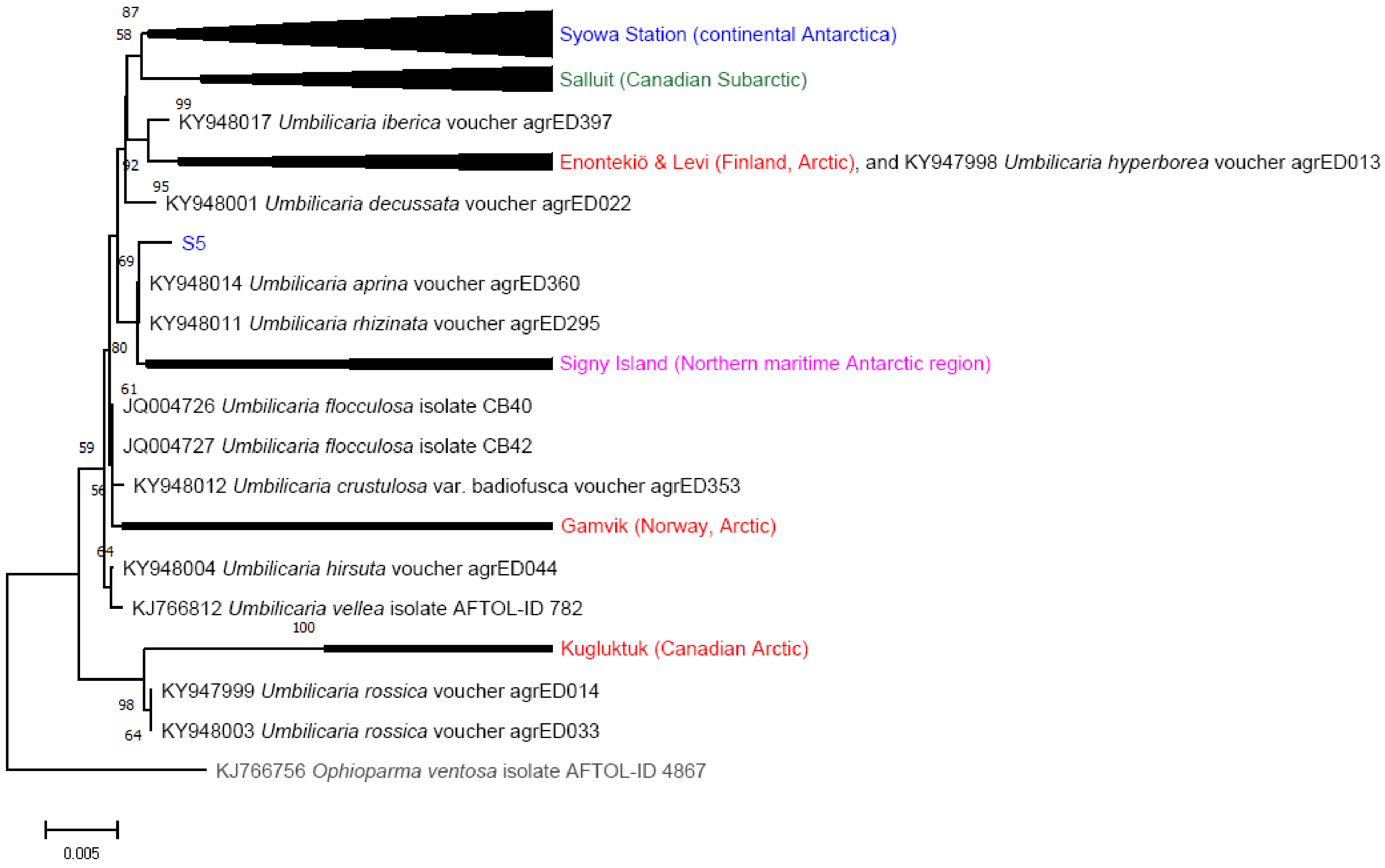


Fig. S12 Phylogenetic tree based on 18S rRNA gene sequences of the rock-dwelling *Umbilicaria* lichens collected from Arctic (red) / Subarctic (green) zones (this study), Antarctica zones including Syowa Station (blue) and Signy Island (pink), each closest species (black, italic) by BLAST search and outgroup (gray, italic) (He et al. 2022). The tree was constructed by Neighbor-Joining method (provided by MEGA-X). The reliability of the inferred tree was tested by 1000 bootstrap replications, and the numbers at nodes demonstrate the bootstrap support values (values < 50 not shown). Lengths of the aligned sequences were around 1.7 kb.

**References**

He Z, Naganuma T, Nakai R, Imura S, Tsujimoto M, Convey P (2022) Microbiomic Analysis of Bacteria Associated with Rock Tripe Lichens in Continental and Maritime Antarctic Regions. J Fungi 8:817 https://doi.org/10.3390/jof8080817
